# Multiscale Analysis of Surface Texture Quality of Models Manufactured by Laser Powder-Bed Fusion Technology and Machining from 316L Steel

**DOI:** 10.3390/ma14112794

**Published:** 2021-05-24

**Authors:** Damian Gogolewski, Tomasz Bartkowiak, Tomasz Kozior, Paweł Zmarzły

**Affiliations:** 1 Department of Manufacturing Technology and Metrology, Kielce University of Technology, 25-314 Kielce, Poland; tkozior@tu.kielce.pl (T.K.); pzmarzly@tu.kielce.pl (P.Z.); 2 Institute of Mechanical Technology, Poznan University of Technology, 60-965 Poznań, Poland; tomasz.bartkowiak@put.poznan.pl

**Keywords:** surface texture, multiscale analysis, additive manufacturing, SLM, 316L

## Abstract

The paper presents the results of tests aimed at evaluating the surface textures of samples manufactured from material based on 316L stainless steel. The analysis of the surface topography was conducted based on the classical approach in accordance with the current standard and with the use of multiscale methods; i.e., wavelet transformation and geometric via curvature. Selective laser melting 3D printing technology was used to produce samples for surface testing. Furthermore, additional assessment of surfaces created as result of milling was conducted. Statistical research demonstrated a differentiation in the distribution of particular morphological features in certain ranges of the analyzed scales.

## 1. Introduction

Additive manufacturing technologies, despite being invented in the 1980s, are not sufficiently well known and refined yet. However, this is not due to the small number of studies conducted, but rather the dynamically developing market of new technologies and materials. Currently, 3D printing has evolved to a degree that a new discipline referred to as 4D printing [1,2,3] is being developed. This emerging field combines the advantages of 3D printing with a modern “smart” structure and materials, enabling minimization of manufacturing time and creation of objects with unprecedented properties, such as shape memory (SMA); e.g., magnetic (SMM) or temperature memory (SMT). The properties of such materials are known for cast alloys such as NiTi, CuZnAl, and CuAlNi; however, in the case of additive technologies with a high degree of anisotropy and properties dependent on the printing direction, studies of such materials seem to be necessary. The creation of composite materials seems to be a very prospective application of 3D printing, either through the use of composite-based materials or via printing on existing objects. The results of imaging testing conducted on the printing of existing objects or via the use of the electrospinning technology to build composite models are presented in [4,5,6]. The 3D/4D printing technologies are currently using three main types of materials when considering the division in terms of the input material form. In the case of layered technologies, the input material can be supplied in the form of a liquid, solid, or powder. When analyzing the available scientific literature and observing the industrial market demand, it can be stated that the technologies utilizing metal-based powder materials are one of the most promising and quickly developing 3D printing applications. This is related especially to a wide range of materials used and their very good properties. Many methods can be currently classified as 3D printing powder technologies utilizing metal powder-based materials. The ISO/ASTM52900–15 international standard [7] specifies the terminology concerning the additive methods and the technologies classified as 3D printing laser methods that allow for melting or sintering of metal powders. The most often used include: selective laser sintering (SLS), selective laser melting (SLM) [8,9], and laser engineering net shaping (LENS). The selective laser melting technology, which was used in this study to build physical sample models, is one of the most popular powder-bed metal-additive technologies allowing for the production of geometrically complex models from steel-based materials, such as: stainless steel 316L and17–4PH, maraging steel M300, aluminum alloys AlSi10Mg and AlSi7Mg, Nickel 718, Nickel 625, Titanium Ti6Al4V ELI Grade 23, and Cobalt CoCrMo [10,11]. In addition, tool wear [12] during the machining process is highly dependent on the material and its properties, which in the case of 3D printing, especially with metal powder-based materials, can cause excessive cutting-edge wear. As demonstrated by multiple studies, the quality of the technological surface layer and the strength of model manufactured using the SLM technology depend on many technological parameters, such as laser scanning speed and power, layer thickness, building chamber temperature, protective atmosphere, and the printing direction. Due to the layered nature of model building, it is the layering direction referred to as the printing direction (Pd) that seems to be an essential technological parameter when analyzing the dimensional and shaping accuracy, the technological quality of the surface layer, and the surface microstructure. Studies on the technological surface layer of models manufactured using the SLM technology and other laser technologies with consideration of the technological parameters’ impact are presented in many research papers [13,14,15,16,17,18,19]. Calignano et al. [16] used the Taguchi statistical method to assess the impact of technological parameters on the surface layer’s quality. The paper features an analysis of the laser speed and hatch distance, as well as designation according to which a reduction in the melted surface’s laser beam scanning speed is advantageous to the surface layer’s quality. Read et al. [17] analyzed the impact of technological parameters, such as laser speed, hatch distance, laser power, and model position on the building platform (in two planes), on the manufactured models’ porosity. The critical energy density was designated in the paper, giving a minimum pore fraction amounting to 60 J/m^3^. Hitzler et al. [18] mainly analyzed the impact of the laser speed and power, hatch distance, and energy density on the surface roughness expressed as parameter Ra. Furthermore, the samples were positioned in different variants on a platform in relation to planes X-Y; i.e., the printer working table. Calignano [19] analyzed the surface texture and accuracy of samples produced by laser powder bed fusion technology. It was shown that the choice of parameters of conversion from the CAD model to STL file and the setting of process parameters can affect the accuracy. Moreover, it was stated that the surface roughness was mainly caused by the process parameters and building orientation.

In the case of finishing of parts manufactured using 3D printing from metals, publications mainly concern the milling of the manufactured models [20,21,22]. In [20], the authors presented the results of testing the surface texture of SLMed samples made from AlSi10Mg powder with the use of a TruPrint 1000 printer. After manufacturing using the additive technology, the models underwent milling, and then their surface roughness was measured to quantify the Ra and Rz parameters. The test results demonstrated a 20% improvement in the models roughness after milling when compared to the roughness immediately after printing. Furthermore, the paper featured an analysis of the impact of the SLM 3D printing technological parameters on the quality of the built surface. In [21], the authors tested parts manufactured from Titanium Ti6Al-4V powder-based material using Wire Arc Additive Manufacturing (WAAM). Then, they also analyzed the surface quality and the samples’ tensile strength. They demonstrated that the mechanical properties of the manufactured models were very high, and met aeronautical standards. Furthermore, no differences were demonstrated in the strength of the models cut from sample models at various angles, which corresponded to the models manufactured at other set printing directions. The paper also featured an analysis of the influence of the printing parameters on the surface quality; e.g., pockets. In [22], the authors studied the impact of milling on the surface quality of models manufactured from Ti6Al4V titanium powder using SLM technology. The paper features a comparison of the obtained results with results for samples obtained with conventional manufacturing technologies. The analyzed parameters concerned both 3D printing and milling, including their impact on the quality of the manufactured surface (Ra), hardness, and microstructure. It was shown that the samples manufactured with the SLM technology demonstrated greater hardness after milling than samples manufactured with conventional manufacturing methods and then processed mechanically.

There is an abundance of methods to describe the topographies of engineered surfaces. The most common way is to characterize measured texture by ISO 25178–2 areal parameters [23]. These characterizations focus on the properties of an entire analyzed region and are based on statistical measures like mean, standard deviation, or root mean square of heights, slopes, area, volume, or curvatures. The most basic height parameters were originally developed to study conventionally manufactured surfaces; i.e., formed by machining or other subtractive technologies. However, in the case of metal additive manufacturing, the geometric complexity of the created surface topographies failed to be captured by standard characterization approaches [24,25]. A potential remedy would be to describe process-specific topographic features that are inherent to their fabrication, such as pits and hills, ridges, and valleys, which are often present in the microscopic images of additively manufactured metal parts [26]. Some feature-based parameters, included in the ISO standard, are potentially relevant for characterization of the formations created by AM, but they are limited only to hills and dales. More general feature-based characterization of laser powder bed fusion was shown by Senin et al. [25], and for electron powder bed fusion by Newton et al. [27]. Those involved characterization of other topographic features that can be decomposed and described separately by applying a pruning algorithm. However, they did not provide an insight into the scales of those features. This becomes an important aspect, as topographic features of a particular size are best discernible when observed and analyzed at particular scales. This is the very essence of multiscale methods, which were successfully applied to study the additively manufactured surface topographies [28,29]. The scale is an important factor to be considered, as physical interactions between the manufacturing process and formed surfaces can occur at multiple scales during fabrication. In terms of additively manufactured surfaces and multiscale analysis, only geometric approaches were noted. This includes area-scale methods [30,31] and curvature tensor estimation for multiple scales [29]. Other multiscale methods that could be potentially applied to study the complexity of AM textures include sliding bandpass filtering, structural function, and wavelet transform [28]. The last one is particularly interesting, as it has a wide spectrum of mother wavelets that can be selected based on the specific nature of the information that is being sought. It is particularly important in the case of additively manufactured surfaces that are characterized by irregular distribution of surface irregularities in particular directions. Furthermore, the method allows both the detection of defects formed on the surface and the identification of their locations. Individual, untypical surface features, which are common in AM, are highlighted in particular scales of such analysis, which is an advantage in comparison with classical concepts of surface evaluation. Studying the multiscale effects of the finishing on the resulting surface topography of additively manufactured parts is not possible when using traditional characterizations. Therefore, scale-dependent methods appear to be of great potential to capture and understand the nature of changes in additively manufactured surface textures made by postprocessing. This paper addresses that issue.

As we observed, publications concerning machining of AM parts focus mainly on the milling of models manufactured by 3D printing, and unfortunately mainly concern a narrow range of roughness parameters (usually Ra and Rz). Due to the above and the geometric complexity of 3D printed surface topographies, the present study, which focuses on the impact of the printing direction and subsequent milling on the quality of the surface texture, seems to be justified and required for a full understanding of the 3D printing and machining processes. The analysis covers an in-depth evaluation of profile (2D) and areal parameters (3D), and it is accompanied by two multiscale analyses: wavelets and curvature. The latter is especially important to determine the scales with the strongest interactions between the processing and the obtained surfaces.

## 2. Materials and Methods

### 2.1. Sample Preparation

**Figure 1:**
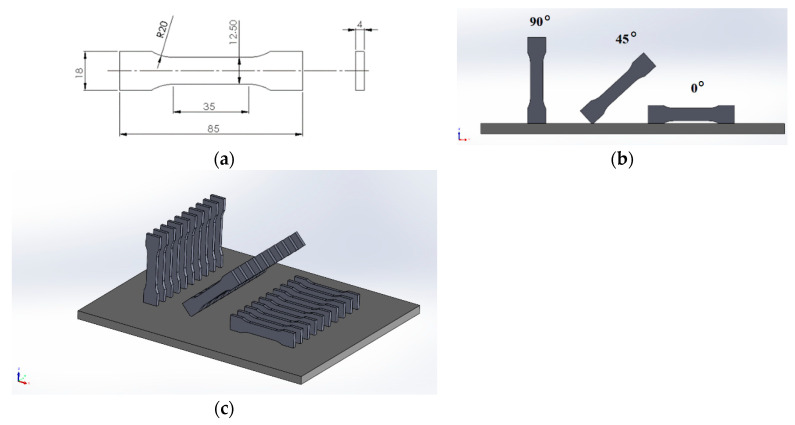
Sample placement on the 3D printer platform: (**a**) dimensions, mm; (**b**) 2D-view; (**c**) isometric view.

The test samples were manufactured by selective laser melting (SLM), and utilized 316L steel (chromium–nickel–molybdenum austenitic) powder (GE, Boston, MA, USA), with a grain size below 30µm. The chemical composition and mechanical properties as declared by the manufacturer are presented in Table 1 and Table 2. The model was built with the use of the CONCEPT LASER M2 machine (GE, Boston, MA, USA) [32]. This machine has a working platform of 250 × 250 × 350 mm^3^ (x, y, z) and a laser system of 2 × 200 W. The machine allows using various materials, including 316L, CL 30/31AL, titanium, bronze, and nickel-based powders, which are common, especially for dentistry applications. Upon completion of the manufacturing process, the samples were subjected to an annealing (heated in a furnace to 550 °C for 3 h and then soaked for 6 h to cool down). The geometry of sample was designed in SolidWorks software as presented in Figure 1a. The samples were positioned on the building platform in three different orientations with respect to the X–Z plane; i.e., 0°, 45°, and 90° (Figure 1b,c). Ten samples of each orientation group were prepared (a total of 30 samples).

**Figure 2:**
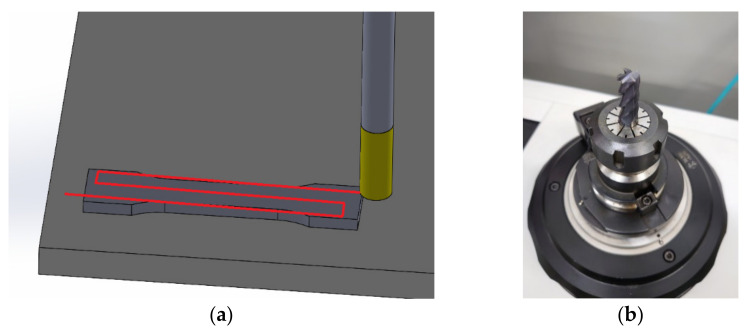
(**a**) Milling strategy for printed samples; (**b**) milling cutter.

The next stage was postprocessing by milling with the use of a Hermle B300 machine (Gosheim, Germany). Due to the high cutting forces that could be generated by the milling head, a smaller cutter was chosen. The milling was performed on the samples’ side plane as presented in Figure 2a. The machining parameters were as follows: rotation speed *n* = 1000 rev/min, feed f_z_ = 0.02 mm/tooth, machining depth of cut a_p_ = 0.1 mm, overlap of surfaces h = 3 mm. A four-tooth end mill (fine grained sintered carbide) with a 10 mm diameter was used as a cutting tool (Figure 2b).

### 2.2. Measurements

The postprocessed samples underwent an assessment of the quality of the obtained surface textures. The surface morphology analysis was conducted with two objectives. The first was to compare the surface textures of as-built and as-machined samples created by SLM. This analysis allowed us to determine the usefulness of performing finishing via milling for additively manufactured surfaces. The second studied effect was the built angle on the pre- and postprocessed textures. A Talysurf CCI Lite optical profilometer was used to measure surface topography. It is based on coherence correlation interferometry, as in the Miaru interferometer. The measurements were taken using 10× magnification, which resulted in measuring an area of 1.66 × 1.66 mm^2^, which was represented by the 1024 × 1024 point matrix. Ten independent measurements in different regions were taken for each printing direction. No outliers were noted.

### 2.3. Surface Topography Analysis

#### 2.3.1. Conventional Analysis with ISO Standard Parameters

In addition, as part of complex testing of the samples’ surfaces, the surface topography parameters were analyzed in accordance with the ISO 25178 standard. The shape was removed by using the second-degree polynomial LC, the surfaces were leveled, and Gaussian filters were used. The cut-off wavelength was λc = 0.8 mm. Four groups of spatial parameters were used in the analysis. The results for the selected parameters are presented herein, whereas the results for all of the analyzed parameters are presented in the Supplementary Materials in the form of box-and-whisker plots. The height parameters are most often used for quantitative surface analysis. They enable the estimated specification of the nature of surface irregularities. The areal feature parameters are a group of parameters that allows identification and characterization of individual peaks and pits. Furthermore, the parameters from this group can be used to assess the density of their occurrence. The functional parameters are parameters calculated based on the Abbott–Firestone curve, and can especially be used for assessing the tribological properties of machine parts that are in contact; e.g., roller bearings. The spatial parameters form a group that describes the orientation of the given surface. They can be used to determine the surface isotropy or anisotropy. Detailed descriptions of particular roughness parameters can be found in the Nomenclature and Abbreviations section.

#### 2.3.2. Multiscale Curvature Tensor Analysis 

In this study, we estimated components of curvature tensor **T**, which can be noted as a product:

(1) T=D·P·D

where $P=\;\left(k_{1},k_{2},n\right),$ and:

(2) $$D=\left[\begin{array}{ccc} \kappa_{1} & 0 & 0\\ 0 & \kappa_{2} & 0\\ 0 & 0 & 0 \end{array}\right]$$

where *κ*_1_ and *κ*_2_ are the eigenvalues that refer to the maximum and minimum principal curvatures, respectively. The eigenvectors **k**_1_, **k**_2_ correspond to principal directions of maximum and minimum curvature, while **n** is the surface normal at the location of the calculated curvature. Mean (*H*) and Gaussian curvature (*K*) were calculated from principle curvatures.

Data from areal surface measurements are usually a discrete set of point coordinates with regular spacing in the x- and y-direction. In this case, calculation of the constituents of the first and second fundamental forms requires either using discrete differences instead of differential operators or fitting a continuous surface. In this study, we used a modified Theisel’s approach to estimate curvature tensor, which involved triangulation of the manifold and assumed the height and normal vector functions to be linear on every triangle; i.e., piecewise linear on the manifold. A detailed explanation of how curvature tensor can be calculated for each scale can be found in [33].

The geometric approach in multiscale analysis is based on the idea that the shape of topographic features, as well as their other measures (area, length, radius of curvature), depend on the scale of observation. At finer scales, surfaces appear as a mosaic of steep-sloped details of high curvature, while at coarse scales, only waviness or form can be discernible. The geometry of features created by machining and those that resulted from laser sintering should manifest themselves at ranges of scales associated with their sizes. This was studied here by calculation of the maximum, minimum, mean, and Gaussian curvature at each location for range of scales between 1.623 and 107.108 µm.

Since the constituents of curvature tensor could be determined at each location, statistical measures were introduced to quantify their distributions as a function of scale. Average and standard deviation measures were considered as defined in [34,35]. A list of the parameters with their short description is presented in Table 3.

#### 2.3.3. Wavelet Transformation as Multiscale Analysis

Another multiscale method used in surface metrology to characterize surface texture is wavelet transform. Having analyzed the state of the art, it can be stated that wavelet transform is used in diagnosis of surfaces or their assessment. Modern filtration methods allow decomposition of the signal into components with various frequency bands and then assessment of the given signal in its specific range and at an adequate scale. The signal filtration in the discrete approach is based on using adequate filters; i.e., the high-pass and low-pass filters. Therefore, a specific iteration and combination of such transformations can lead to obtaining a signal with clear visibility of the signal’s features and its locations, which are not visible during filtration with other data-processing algorithms. Wavelet transform is used as an alternative for the common Gaussian filter to determine the surface roughness or waviness [36], but also for surface diagnosis, 2D and 3D signal feature specification [37,38], processing such as milling or turning [39,40], or a measurement process; e.g., contact profilometry [41].

The key issue here is the correct determination of the transform parameters. Properties of a particular wavelet should be taken into account, as they affect the results of the analysis [42,43]. Particular wavelet groups differ between one another in terms of the support length, smoothness, symmetry, or orthogonality, which makes the filtered signal differ at subsequent levels of analysis for various mother wavelets [44]. With the use of the wavelet transform, it is therefore possible to conduct a complex assessment or classification of the surface texture obtained with the use of a series of manufacturing techniques, or tested with the use of various advanced measurement methods. In the continuous approach, the analysis process is based on using the locally determined function (wavelet), which is adequately located in time through shifts and in frequency through scaling. To describe high-frequency information, the mother wavelet is scaled and compressed by using the small-scale parameter values. To describe low-frequency information, the wavelet is stretched, while the scale coefficient value and wavelength are longer. Such a scaled wavelet is compared with the analyzed signal and then shifted by a specific distance and again compared with the input signal. The coefficients obtained this way allow for identifying the certain features of the signal for the determined scale and detecting specific features in them. Studies covering continuous wavelet transformation (CWT) were conducted, among others, by Brol and Grzesik in [45]. The authors used CWT in the diagnosis assessment of turning by analyzing the roughness profiles. They showed the method was especially useful for assessing random profile changes. In another study, the authors analyzed the pavement texture during polishing with the use of CWT [46]. They demonstrated that it can be successfully used to analyze the texture of aggregates used in pavement composition. The authors designated the RMS parameter (root mean square) for each of the analyzed scales. In addition, Bruzzone demonstrated that the wavelet approach provides a powerful multiscale representation of the rough surfaces [47]. In [48], the authors quantified the effect of chipping in the ceramic insert on the surface profile signature of the workpiece. In [49], Chandra and Sekhar compared the selected signal-processing methods to assess their detection performance. They showed that the continuous wavelet transformation was more preferred for analyzing noisy signals.

The 2D Mexican hat mother wavelet was used in this study. The wavelet is defined as the second derivative of the Gaussian probability density function (3). It is an isotropic, radial, and symmetrical wavelet that allows the assessment of the distribution of signal extremes. It has good localization properties both in terms of space and frequency. It checks the conditions of existence well, and has a filter’s bandpass behavior [50].

(3) $$\psi \left(x,y\right)=-2\pi {\left(x^{2}+y^{2}\right)}^{p/2}e^{-\frac{{\left(\sigma_{x}x\right)}^{2}+{\left(\sigma_{y}y\right)}^{2}}{2}}$$

In this research, parameter values were assumed: *p = 2, σ_x_ = σ_y_ = 1,* therefore the wavelet assumes the following form (4):

(4) $$\psi \left(x,y\right)=-2\pi \left(x^{2}+y^{2}\right)\;e^{-\frac{\left(x^{2}+y^{2}\right)}{2}}$$

### 2.4. Discrimination Analysis by Two-Way ANOVA

From the quality-control perspective, it is important to be able to differentiate or distinguish between surfaces that perform, were manufactured, modified, or treated differently. This ability is called discrimination, and thanks to multiscale analysis, it becomes possible to identify which surface-characterization techniques, relating parameters and at what scales, are the most convenient at discerning topographies. The idea comes from the fact that topographic features of certain sizes and shapes that are signatures of particular manufacturing processes can be best discernible at a certain scale or scales of observation.

This study focused on the effect of conventional machining on the surface topography created on additively manufactured metal parts printed at different orientations. To determine if the effect was statistically relevant, for each surface characterization, the parameter *p*-value was calculated using two-way ANOVA and presented for each scale. Build orientation and machining (yes or no) were considered as two independent variables. The ability to discriminate surfaces with 95% or greater confidence was considered as sufficient (*p* < 0.05). Shapiro–Wilk tests were used to test for normal distributions of residuals.

### 2.5. Technological Inheritance

One of the main assumptions of each technological process is the striving for obtaining products with satisfactory quality with simultaneous minimization of the manufacturing costs. The use of the additive technologies allows in most cases for manufacturing finished products, which is especially important in the case of prototype manufacturing. A proper selection of the 3D printing technological parameters (printing direction, laser power, material layer thickness) allows controlling the manufacturing process to obtain products with desired dimensional and shape accuracy, surface layer quality, and satisfactory mechanical properties [13]. However, in some cases, this was insufficient, and the parts that were manufactured additively required additional finishing processes. Due to the above, it was necessary to perform treatment aimed at optimizing the 3D printing technological parameters to obtain products with the assumed accuracy with simultaneous minimization of additional operations.

The technological inheritance concept can be used to optimize the 3D printing technological parameters [51,52,53]. The phenomenon of transfer of particular object features from one technological process to another is called technological inheritance, whereas if the features remain permanently in the final product, the phenomenon is referred to as technological heredity. If the inherited features affect the given object’s operating properties, then we are dealing with operational heredity [54]. An analysis of the technological inheritance of 3D printing and milling of elements manufactured using the SLM technology will allow for determining the impact of the selected technological parameters on the quality of the elements’ surface layer. Then, it should be possible to introduce certain corrections to the designing stage of the technological process to obtain products that meet specific requirements.

## 3. Results

### 3.1. Measurements

**Figure 3:**
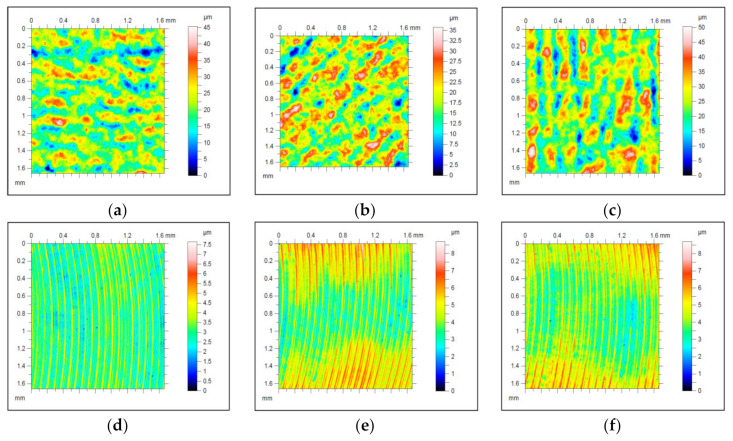
Representative images of surface topographies prior to processing built at (**a**) 0°, (**b**) 45°, and (**c**) 90°; and after processing at (**d**) 0°, (**e**) 45°, and (**f**) 90°.

The surface topography measurements conducted in an optical profilometer allowed for the qualitative and quantitative assessment of the irregularities formed on the samples’ surfaces. Figure 3a,c presents selected topographic images for samples manufactured additively, with consideration of the analyzed building angle, while Figure 3d,f presents selected images for these samples after face milling. When analyzing the obtained images, it was possible to notice that the surface prior to processing featured a surface orientation that was analogous to the addition direction. The surfaces consisted of a directionally located series of peaks and pits in accordance with the laser scanning direction. The created staircase effect was visible in all of the analyzed surfaces; however, the size of the irregularities was not the same in each path. This was related to the material’s flow and solidification during building and the layer placement direction, which clearly pointed to the anisotropy of the properties of additively manufactured models. When conducting a qualitative assessment of the images after processing, it must be stated that milling removed these surface features and left regular processing marks, which are typical for this type of processing. The height of the irregularities was reduced by an order of magnitude. The surface images after processing demonstrated microcavitation, which presumably was an effect of breakaway of powder that was not completely melted. Furthermore, when analyzing the stereometry of the surface printed at angles of 45° (Figure 3e) and 90° (Figure 3f), it was possible to notice an uneven removal of material by the milling cutter. The specific surface geometry area visible in the samples’ central part was shaped due to the elastic deformations formed during the milling operation. Then, the material was cut most effectively in the central part of the sample. The images of surfaces after processing also featured visible local material losses caused by the laser and powder that was not completely melted. These chipping locations occurred on the side of pits mapped by the cutting tool’s geometry near their peaks. The dimensions, distribution, and frequency of these losses seemed to be independent from the printing angle.

### 3.2. Analysis of Surface Topographies

**Figure 4:**
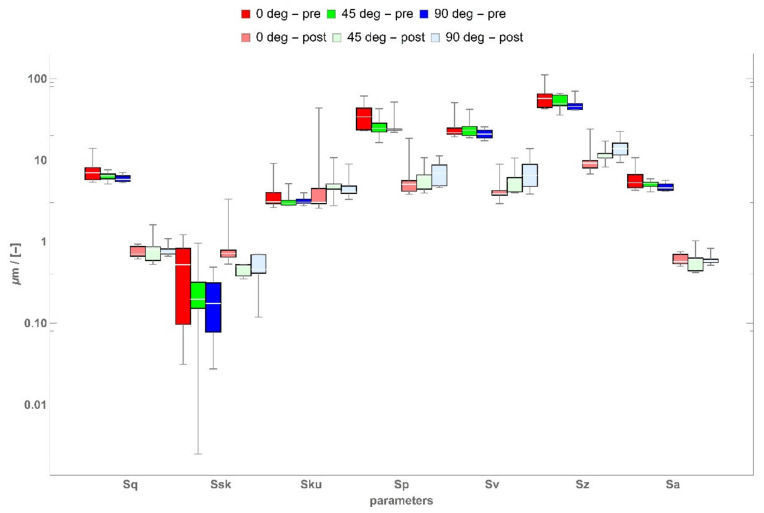
Height areal parameters.

The overview of the results of selected areal parameters that are most often used in industrial practice designated in accordance with the ISO 25178 standard is presented in Figure 4 in the form of a box-and-whisker plot. Further visualizations for other areal field parameters can be found in the Supplementary Materials.

When analyzing the topography parameters from height group, specifically the arithmetic mean height Sa and the root mean square Sq, it was possible to notice a clear impact of the printing direction on the parameters’ values. Both for Sa and Sq, the highest values were obtained for the printing angle of 0°, whereas the lowest values were obtained for the printing angle of 90°. Furthermore, the printing of samples positioned at the angle of 0° in relation to the building platform resulted in a higher dispersion of the roughness parameters Sa and Sq in the tested samples. The milling caused a reduction of Sa and Sq, which was anticipated. Similar conclusions can be made when analyzing the maximum height; i.e., Sz. The lowest value of parameter Sz was also obtained for samples manufactured at the angle of 90°. The milling also led to a decrease of Sz for all three build angles. On the other hand, when referring to the surface topography height distribution skewness Ssk, it can be stated that samples printed at the angle of 0° featured the highest dispersion of the measurement results. Furthermore, the use of a milling sample caused an increase in the values of parameter Ssk for all of the analyzed printing directions. The skewness was positive for all of the tested samples, both prior to and after processing. This means a large number of singular sharp peaks occurred on the tested surfaces, which is clearly visible in Figure 3. This nature of irregularities also was confirmed by the kurtosis, in which Sku > 3.

### 3.3. Wavelet Analysis

**Figure 5:**
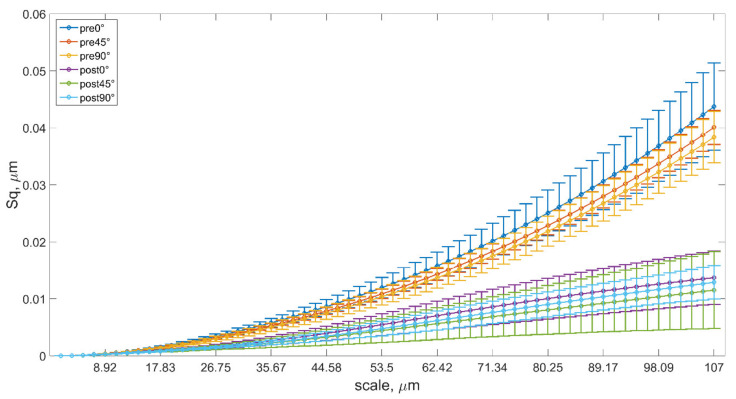
Evolution of root mean square height (Sq) with scale. Please note that “pre” and “post” terms refer to postprocessing via machining.

The signal assessment with the use of the wavelet transform was aimed at analyzing the number of irregular, sharp peaks and pits, distribution of extremes on the sample surfaces with consideration of the impact of the build angle, as well as postprocessing via machining. The 2D Mexican hat wavelet was used to allow for the assessment of such surface irregularities, in particular intensity scales. The assessment was conducted mainly with the use of the root mean square height (Sq) coefficient, which is widely used to assess signals formed as result of the wavelet decomposition of various types of surfaces [44,46]. In addition, the results for other selected coefficients with which the 3D surfaces could be described, depending on the needs, are included in the Supplementary Materials to provide a broader view on the analysis process. For this purpose, the arithmetical mean height (Sa), skewness (Ssk), and kurtosis (Sku) parameters were selected. The results of root mean square calculated for all three groups are presented in Figure 5. The scale coefficient was designated based on the effective support width.

The use of the wavelet transform allowed for the assessment of the surfaces in multiple scales. When analyzing the data presented in the above chart and the data provided in the Supplementary Materials, it was possible to reach conclusions on the quality and magnitude of extremes that occurred on the sample surfaces. Our study demonstrated that there was a distinction in such peaks and pits, depending on the model-building angle and the machining effect. This was especially visible for higher-scale values, where higher Sq values were obtained, thereby pointing to a higher intensity of the distribution of extremes in terms of quantity and value. It can be noted that there is a substantial variation in the topography of such sample surfaces prior to and after processing. The statistical testing confirms the deliberations considered based on Figure 5. The entire range of the analyzed scales showed the statistical distinction of parameters Sa and Sq when considering the model-building angle and machining effect as separate and independent factors. This means that when analyzing surfaces through these values, it is possible to state that they were not substantially similar to one another. For other assessed parameters, the distinction was only recorded for a certain range of the analyzed scale. When taking into consideration the model-building angle as an independent factor, the parameter Ssk did not allow for the statistical distinction of the surfaces for a majority of the scales’ range, aside from scales higher than 85 µm, whereas when taking into consideration the machining effect as an independent factor, the parameter Ssk allows for the statistical distinction of the surfaces for a majority of the scales range, aside from scales higher than 46–59 µm. When assessing the values of this coefficient, it must be stated that the surfaces obtained as result of machining featured local pits, while surfaces prior to processing featured local peaks. Analogous deliberations could be considered for the kurtosis parameter. Aside from some borderline exceptions, the parameter did not allow for the distinction of the surfaces in terms of the model-building angle and machining effect when considered as separate and independent factors. However, when taking into account both factors, the parameter Sku was best at distinguishing the surfaces between one another. Other parameters allowed for distinguishing the surfaces only for a narrow scale range. Considering build angle and processing combined, surfaces could be discriminated for a narrow range of scales (<19.613 µm, 21.396 µm>), which corresponded to feed per tooth f_z_ during machining. Detailed results of the statistical analysis for all scales and parameters using two-way ANOVA can be found in the Supplementary Materials.

It can be concluded that there was no single versatile parameter that would allow for the distinction of the surfaces in terms of these effects jointly or separately for the purpose of assessing the local extremes of peaks and pits. It seems that the topographies formed as result of using the additive technologies and milling differed, but they had surface features that were statistically similar in certain scales.

### 3.4. Multiscale Curvature Analysis 

**Figure 6:**
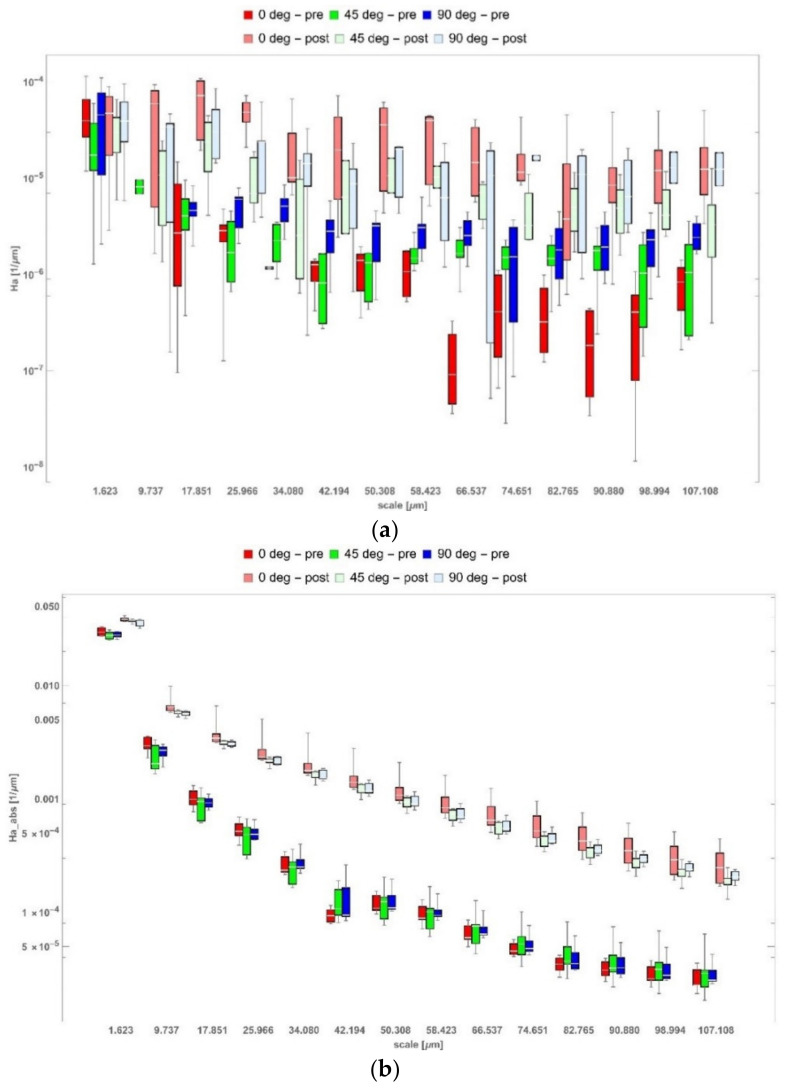
Evolution of selected curvature parameters with scale: (**a**) average mean curvature—Ha; (**b**) average absolute mean curvature—Ha_abs_. Please note that “pre” and “post” terms refer to postprocessing via machining.

The ability to discriminate between surfaces changes depending on the parameters and scale. Differentiating versus machining (whether it was performed or not) was possible for all curvature parameters, apart from average mean curvature (Ha), as presented in Figure 6a, and narrow ranges of analyzed scales for average Gaussian curvature (<24.343 µm, 42.194 µm>), average maximum curvature (=14.606 µm or ≥ 58.423 µm), and a few scales for Kqabs. Absolute parameters that did not consider the sign of curvature performed better in discriminating than their signed counterparts. Average absolute maximum curvature is a characterization parameter that exhibits superior performance in differentiating versus machining and build orientation independently for all analyzed scales at *p* < 0.05 (Figure 6b). Some parameters allowed us to differentiate versus build angle only for a narrow range of scales (s); e.g., κ2a for s ≤ 34.080 µm and κ2q for s ≤ 50.308 μm and s ≥ 107.108 μm. There was group of parameters that did not allow discrimination versus build-up angle for medium scales; i.e., between 17.851 and 34.080 μm, including κ1a, κ1q, Ha, Ka, Kq, κ1q_abs_, Hq_abs_, Ka_abs_, and Kq_abs_. Minimum principal curvature and corresponding parameters could be used to differentiate between surfaces for larger scales; i.e., between 58.423 μm and 82.765 μm. This meant, as in the wavelet analysis, that there was no universal metric that could be used in all applications to confidently discriminate between topographies. The surface morphology created by a certain fabrication process requires individual parameters to capture all the important aspects of its geometry that resulted from the manufacturing. This became evident when considering build orientation and postprocessing together. In that case, the surface topographies could be discriminated for finer scales with minimum principal curvature: κ2a, κ2q, κ2a_abs_, κ2q_abs_, and Ka_abs_. For coarse scales, this was possible for maximum and mean curvature (excluding Ha). The detailed results for all scales and parameters using two-way ANOVA can be found in the Supplementary Materials.

The effect of postprocessing via machining was seen in the increase of the topographic curvature from 10-fold for principal and mean to 1000-fold for Gaussian. This meant that the surface morphology had been dramatically changed by the postprocessing. Additive manufacturing created directional features aligned with the laser scanning path, which resembled a composition of round or sinusoidal hills and valleys. When compared to the as-machined topographies, the maximum height was circa six times higher, but this effect was not reflected in the higher curvature for the as-build samples. This could be explained by looking at the geometric features created by postprocessing. Each of the machined surfaces was composed of circular valleys formed by the circular tool nose tip. These valleys were separated by narrow, sharp, and steep-sloped ridges with high curvature. These geometric features in particular contributed the most to the increased value of curvature parameters noted for the postprocessed samples. More subtle effects were seen when considering the effect of build orientation on the resulted surface topographies. Before the machining, the highest curvature was generally observed for surfaces built at 45° orientation, with the other two groups showing similar results. Postprocessing changed that tendency, making the 0° build orientation of the highest curvature. For that group, the results for machined samples seemed to be the most dispersive, which potentially meant that the postprocess was the least stable for that input material.

### 3.5. Analysis of the Technological Inheritance of the Additively Manufactured Surfaces after Milling Process

One of the main assumptions for the analysis of the manufacturing processes’ inheritance is the determination of the technological parameters’ impact on the formation of specific surface features that can then affect the subsequent technological process operations. The conducted testing featured an analysis of the impact of the printing direction, which is one of the most important parameters of additive technologies, on the formation of the printed surfaces’ stereometry. Based on the conducted testing, it can be stated that the printing direction had a substantial inheritable impact on the printed surface quality. When assessing the surface roughness quantified with parameters Sa and Sq, it can be stated that the printing angle of 90° allowed the formation of surfaces with the lowest roughness. Milling, which introduced additional inheritable factors to the final element of the machining parameters, was performed to reduce and change the roughness structure of the printed surface. It caused the roughness reduction and obtained a specific surface orientation that corresponded to the cutting marks. It would seem that if the same tool and the same cutting parameters were used, the milled surface structure for samples printed at three different angles should be the same. However, for surfaces printed at 45° and 90°, the samples demonstrated a trend of elastic deflection during milling, resulting in a specific structure in the sample’s central part. This phenomenon did not occur for samples printed at 0°. This could point to a higher rigidity of the samples positioned this way on the printing platform. When summarizing the inheritance of the milling of additively manufactured surfaces, it can be stated that the technological parameters of 3D printing were important inheritable factors that affected the subsequent processing operations.

## 4. Discussion

There is no single universal characterization that can fully capture the complexity of surfaces created by additive manufacturing. In this study, we focused on the effects of build-up angle and conventional machining on the resulting texture of 3D printed stainless steel. This was analyzed by the conventional ISO 25178 areal parameters and two multiscale methods: wavelet transform and geometric via curvature. All three characterizations performed well in confidently differentiating between the as-built and as-machined samples. Some exceptions included Std (surface texture direction), which was strongly dependent on how the sample was actually oriented under the microscope while measuring, or was aligned in postprocessing software and average mean curvature (Ha), as well as narrow ranges of scales for average Gaussian curvature (Ka), average maximum curvature (κ1a), and a few scales for Kq_abs_, which appeared to be random.

For curvature, some parameters allowed differentiation versus build angle only for a narrow range of scales (κ2a, κ2q), or only for medium scales (κ1a, κ1q, Ha, Ka, Kq, κ1q_abs_,Hq_abs_, Ka_abs_, Kq_abs_). For larger scales, minimum principal curvature and corresponding parameters could not be used to differentiate between surfaces. Understanding how and at what scales the fabrication process affected the processed material required a group of individual parameters to capture all the important aspects of its geometry that resulted from the manufacturing [24,55]. This stood in opposition to what is commonly used in the industry [56] and scientific community [57], where, for the analysis of geometrically complex surface topographies, basic profile (Ra, Rz, Rq) or areal (Sa, Sq) parameters are used most often. This paper sought to provide some justification for why more sophisticated characterization should be applied in the description of textures created by additive manufacturing.

The research results presented in [13] featured an assessment of the impact of the printing direction on the formation of frontal and side waviness in sample surfaces. When analyzing the results for the frontal surface, it was possible to notice a similar trend as seen in the topography parameters of surfaces obtained in our study. An increase in the printing direction caused a reduction in the parameters determining the arithmetical mean height.

When analyzing the parameter Sz, which determines the maximum roughness height, it was possible to notice an increase in its value along with an increase in the printing angle. The milling caused a reduction in this parameter for all of the analyzed samples manufactured at different orientations in relation to the building platform. However, when considering the maximum roughness height of the surface after milling, it was possible to notice an inverse trend when compared to the surface only after printing. In this case, an increase in the printing direction caused a reduction in the parameter Sz. This could derive from the removal of sharp roughness peaks during milling, which was especially visible for the printing angle of 90°, which featured the highest parameter Sz.

It was possible to notice an upward trend in the next surface parameter, Sv. This was caused by the nature of the machining process, in which the cutting edge left clear marks on the surface in the form of pits cut by the cutting edge. This trend was not recorded for additively manufactured surfaces. The parameter value was similar regardless of the addition angle.

The assessment also covered the distinction and similarity of the surface topography based on the distribution of peaks and pits. Analysis of the distribution of surface extremes utilizing the wavelet transformation demonstrated that there was a substantial distinction between such irregularities, depending on the building angle, processing type, and scale at which they were assessed. It was concluded that it was not justified to use all the parameters described herein to characterize such distributions, because the skewness and kurtosis featured no substantial distinction between particular values for nearly the entire range of the assessed scales. Such deliberations could be considered with the use of Sq or Sa, because it was demonstrated that when the model building angle and the machining effect were considered in the full range of the analyzed scales, the parameters were statistically distinct, and therefore the distribution of peaks and pits was also distinct.

This study had some limitations that have to be addressed in further research work. This concerns mostly the number of different built-up angles. The higher variety of built-up orientations could help to establish functional relations for certain surface-characterization parameters and contribute to more in-depth understanding of how the fabrication process governs the creation of certain topographic features on the surface. In addition, machining was performed at the same unchanged technological parameters. For example, manipulating the feed rate and cutting speed could potentially affect the frequency of micropits caused by removing powder that was not fully melted from the material. This might change the values of the surface-characterization parameters. Feed rate also affects the distance between valleys and thus alters the surface morphology as well. Further research in this direction should be conducted.

## 5. Conclusions

Surface characterization based on the classical approach in accordance with the ISO standard and on the multiscale approach; i.e., wavelet transformation and geometric via curvature, was used in this paper to identify the variation in the distribution of surface irregularities. Testing was conducted to study the changes in the surface topography in the function of the processing type (additive technology and face milling) and the function of surface orientation in relation to the build angle (0°, 45°, 90°). The results showed that the surface characterization, based on its assessment at many scales, could be useful in topography specification, assessment of its similarity, and quantification of the technological parameters impact on its quality. The quantitative and qualitative testing presented in this paper could serve a purpose for a better understanding of the nature of the phenomena occurring during element shaping in terms of their impact on the shaped structure. The approach based on complex topographic assessment with simultaneous use of many methods could provide new viewpoints on surface irregularities.

Some detailed conclusions could be also drawn:

1. There was no single universal characterization that could fully capture all the aspects of surface morphologies created by additive manufacturing.
2. All three studied characterizations (conventional, and wavelet- and curvature-based) generally performed well in confidently discriminating between as-built and as-machined samples.
3. Maximum roughness Sz increased along with an increase in the printing angle. The milling caused a reduction in this parameter for all of the analyzed samples manufactured at different orientations in relation to the building platform. For processed surfaces, an increase in the printing direction of original samples caused a reduction in the parameter Sz.
4. As for wavelet analysis, skewness and kurtosis exhibited no substantial distinction between particular values for nearly the entire range of the assessed scales. Such deliberations can be considered with the use of Sq or Sa, because it was demonstrated that when the model-building angle and the machining effect were considered in the full range of the analyzed scales, the parameters were statistically distinct, therefore the distribution of peaks and pits was also distinct.
5. Printing direction was an important inheritable factor of laser powder-bed fusion technology and significantly affected the formation of surface topography of parts manufactured from 316L steel. In addition, the appropriately selected print direction in additive technology affected further processing processes.

## Figures and Tables

**Table 1:** Chemical composition of the 316L steel [32].

Component	Cr	Ni	Mo	Mn	Si	P	C	S	Fe
Indicative value, %	16.5–18.5	10.0–13	2–2.5	0–2	0–1.0	0–0.045	0–0.03	0–0.03	Balance

**Table 2:** Mechanical properties of the 316L steel [32].

Properties	Unit	90°, Upright	45°, Polar Angle	0°, Horizontal
Yield strength, R_p0,2_	N/mm^2^	374 ± 5	385 ± 6	330 ± 8
Tensile strength, Rm	N/mm^2^	650 ± 5	640 ± 7	529 ± 8
Elongation, A	%	65 ± 4	63 ± 5	63 ± 5
Young’s modulus	N/mm^2^	-	ca. 200 × 10^3^	-
Hardness	HRC	-	20	-

**Table 3:** Curvature statistical parameters used in the study. Please note that SD stands for standard deviation.

Symbol	Description	Symbol	Description	Symbol	Description	Symbol	Description
κ1a	average maximum curvature	κ1q	SD of maximum curvature	κ1a_abs_	average absolute maximum curvature	κ1q_abs_	SD of absolute maximum curvature
κ2a	average minimum curvature	κ2q	SD of minimum curvature	κ2a_abs_	average absolute minimum curvature	κ2q_abs_	SD of absolute minimum curvature
Ha	average mean curvature	Hq	SD of mean curvature	Ha_abs_	average absolute mean curvature	Hq_abs_	SD of absolute mean curvature
Ka	average Gaussian curvature	Kq	SD of Gaussian curvature	Ka_abs_	average absolute Gaussian curvature	Kq_abs_	SD of absolute Gaussian curvature

## Data Availability

The data presented in this study are available on request from the corresponding author.

## Supplementary Materials

The presented graphs are related to Section 3 and extend the results presented with figures for the ANOVA analysis for the evaluated parameters. Furthermore, we present graphs for ISO, wavelet, and curvature parameters described in the paper. The following are available online at https://www.mdpi.com/article/10.3390/ma14112794/s1, Figure S1: Evolution of selected curvature parameters with scale—average mean curvature—Ha. Please note that “pre” and “post” terms refer to post-processing via machining; Figure S2: Evolution of selected curvature parameters with scale- average absolute mean curvature—Haabs. Please note that “pre” and “post” terms refer to post-processing via machining; Figure S3: Evolution of selected curvature parameters with scale—SD of mean curvature—Hq. Please note that “pre” and “post” terms refer to post-processing via machining; Figure S4: Evolution of selected curvature parameters with scale- SD of absolute mean curvature—Hqabs,. Please note that “pre” and “post” terms refer to post-processing via machining; Figure S5: Evolution of selected curvature parameters with scale—average maximum curvature—κ1a. Please note that “pre” and “post” terms refer to post-processing via machining; Figure S6: Evolution of selected curvature parameters with scale—average absolute maximum curvature—κ1aabs. Please note that “pre” and “post” terms refer to post-processing via machining; Figure S7: Evolution of selected curvature parameters with scale—SD of maximum curvature—κ1q. Please note that “pre” and “post” terms refer to post-processing via machining; Figure S8: Evolution of selected curvature parameters with scale—SD of absolute maximum curvature—κ1qabs. Please note that “pre” and “post” terms refer to post-processing via machining; Figure S9: Evolution of selected curvature parameters with scale—average minimum curvature—κ2a. Please note that “pre” and “post” terms refer to post-processing via machining; Figure S10: Evolution of selected curvature parameters with scale—average absolute minimum curvature—κ2aabs. Please note that “pre” and “post” terms refer to post-processing via machining; Figure S11: Evolution of selected curvature parameters with scale—SD of minimum curvature—κ2q. Please note that “pre” and “post” terms refer to post-processing via machining; Figure S12: Evolution of selected curvature parameters with scale—SD of absolute minimum curvature—κ2qabs. Please note that “pre” and “post” terms refer to post-processing via machining; Figure S13: Evolution of selected curvature parameters with scale—average Gaussian curvature—Ka. Please note that “pre” and “post” terms refer to post-processing via machining; Figure S14: Evolution of selected curvature parameters with scale—average absolute Gaussian curvature—Kaabs. Please note that “pre” and “post” terms refer to post-processing via machining; Figure S15: Evolution of selected curvature parameters with scale—SD of Gaussian curvature—Kq. Please note that “pre” and “post” terms refer to post-processing via machining; Figure S16: Evolution of selected curvature parameters with scale—SD of absolute Gaussian curvature—Kqabs. Please note that “pre” and “post” terms refer to post-processing via machining; Figure S17: Feature areal parameters; Figure S18: Functional areal parameters—part 1; Figure S19: Functional areal parameters—part 2; Figure S20: Height areal parameters; Figure S21: Spatial areal parameters; Figure S22: Evolution of arithmetical mean height (Sa) with scale. Please note that “pre” and “post” terms refer to post-processing via machining; Figure S23: Evolution of kurtosis (Sku) with scale. Please note that “pre” and “post” terms refer to post-processing via machining; Figure S24: Evolution of root mean square height (Sq) with scale. Please note that “pre” and “post” terms refer to post-processing via machining; Figure S25: Evolution of skewness (Ssk) with scale. Please note that “pre” and “post” terms refer to post-processing via machining; Figure S26: ANOVA—Curvature parameters- factor 1—build direct; Figure S27: ANOVA—Curvature parameters- factor 2—machining; Figure S28: ANOVA—Curvature parameters- both factors together; Figure S29: ANOVA—wavelet transformation- factor 1—build direct; Figure S30: ANOVA—wavelet transformation—factor 2—machining; Figure S31: ANOVA—wavelet transformation—both factors together.

## Nomenclature and Abbreviations

(a) Height parameters	
Sa	arithmetical mean height
Sq	root mean square height
Sa	arithmetical mean height,
Sq	root mean square height
Sp	maximum peak height
Sv	maximum pit height
Sz	maximum height
Ssk	skewness
Sku	kurtosis

(b) Areal feature parameters	
Spd	density of peaks
Spc	mean peak curvature
S10z	10-point height
S5p	five-point peak height
S5v	five-point peak height
Sda	mean dales area
Sha	mean hills area
Sdv	mean dales volume
Shv	mean hills volume

(c) Functional parameters	
Smr	peak material portion
Smc	inverse areal material ratio
Sxp	peak extreme height
Sk	core height
Spk	reduced peak height
Svk	reduced valley depth
Smr1	peak material portion
Smr2	valley material portion

(d) Spatial parameters	
Sal	auto-correlation length
Str	texture aspect ratio
Std	texture direction

## References

[1] Zolfagharian A., Mahmud M.A.P., Gharaie S., Bodaghi M., Kouzani A.Z., Kaynak A. (2020). 3D/4D-printed bending-type soft pneumatic actuators: Fabrication, modelling, and control. Virtual Phys. Prototyp..

[2] Zolfagharian A., Denk M., Bodaghi M., Kouzani A.Z., Kaynak A. (2019). Topology-Optimized 4D Printing of a Soft Actuator. Acta Mech. Solida Sin..

[3] Tamay D.G., Usal T.D., Alagoz A.S., Yucel D., Hasirci N., Hasirci V. (2019). 3D and 4D Printing of Polymers for Tissue Engineering Applications. Front. Bioeng. Biotechnol..

[4] Kozior T., Mamun A., Trabelsi M., Wortmann M., Lilia S., Ehrmann A. (2019). Electrospinning on 3D Printed Polymers for Mechanically Stabilized Filter Composites. Polymers.

[5] Kozior T., Trabelsi M., Mamun A., Sabantina L., Ehrmann A. (2019). Stabilization of Electrospun Nanofiber Mats Used for Filters by 3D Printing. Polymers.

[6] Kozior T., Blachowicz T., Ehrmann A. (2020). Adhesion of three-dimensional printing on textile fabrics: Inspiration from and for other research areas. J. Eng. Fibers Fabr..

[7] ASTM (2015). ASTM52900-15 Standard Terminology for Additive Manufacturing—General Principles—Terminology.

[8] Cegan T., Pagac M., Jurica J., Skotnicova K., Hajnys J., Horsak L., Soucek K., Krpec P. (2020). Effect of Hot Isostatic Pressing on Porosity and Mechanical Properties of 316 L Stainless Steel Prepared by the Selective Laser Melting Method. Materials.

[9] Wilhelm M., Wissenbach K.D., Gasser A.D. (1996). Shaped Body Especially Prototype or Replacement Part Production. Patent.

[10] Hajnys J., Pagac M., Mesicek J., Petru J., Spalek F. (2020). Research of 316L Metallic Powder for Use in SLM 3D Printing. Adv. Mater. Sci..

[11] Hajnyš J., Pagáč M., Zlámal T., Petrů J., Kousal L. (2018). Stiffness of 316L stainless steel support structures proposed for the SLM process. MATEC Web Conf..

[12] Stavropoulos P., Papacharalampopoulos A., Souflas T. (2020). Indirect online tool wear monitoring and model-based identification of process-related signal. Adv. Mech. Eng..

[13] Kozior T., Bochnia J., Zmarzły P., Gogolewski D., Mathia T.G. (2020). Waviness of Freeform Surface Characterizations from Austenitic Stainless Steel (316L) Manufactured by 3D Printing-Selective Laser Melting (SLM) Technology. Materials.

[14] Kozior T., Bochnia J. (2020). The Influence of Printing Orientation on Surface Texture Parameters in Powder Bed Fusion Technology with 316L Steel. Micromachines.

[15] Maamoun A.H., Xue Y.F., Elbestawi M.A., Veldhuis S.C. (2018). Effect of Selective Laser Melting Process Parameters on the Quality of Al Alloy Parts: Powder Characterization, Density, Surface Roughness, and Dimensional Accuracy. Materials.

[16] Calignano F., Manfredi D., Ambrosio E.P., Iuliano L., Fino P. (2013). Influence of process parameters on surface roughness of aluminum parts produced by DMLS. Int. J. Adv. Manuf. Technol..

[17] Read N., Wang W., Essa K., Attallah M.M. (2015). Selective laser melting of AlSi10Mg alloy: Process optimisation and mechanical properties development. Mater. Des..

[18] Hitzler L., Hirsch J., Merkel M., Hall W., Öchsner A. (2017). Position dependent surface quality in selective laser melting. Mater. Werkst..

[19] Calignano F. (2018). Investigation of the accuracy and roughness in the laser powder bed fusion process. Virtual Phys. Prototyp..

[20] Matras A. (2019). Research and Optimization of Surface Roughness in Milling of SLM Semi-Finished Parts Manufactured by Using the Different Laser Scanning Speed. Materials.

[21] Veiga F., Gil Del Val A., Suárez A., Alonso U. (2020). Analysis of the Machining Process of Titanium Ti6Al-4V Parts Manufactured by Wire Arc Additive Manufacturing (WAAM). Materials.

[22] Milton S., Morandeau A., Chalon F., Leroy R. (2016). Influence of Finish Machining on the Surface Integrity of Ti6Al4V Produced by Selective Laser Melting. Procedia CIRP.

[23] International Organisation of Standardization (2012). ISO 25178-2:2012. Geom. Prod. Specif.—Surf. texture Areal—Part 2 Terms, Defin. Surf. Texture Parameters.

[24] Leach R., Thompson A., Senin N., Maskery I. A metrology horror story: The additive surface. Proceedings of the ASPEN/ASPE Spring Topical Meeting on Manufacture and Metrology of Structured and Freeform Surfaces for Functional Applications.

[25] Senin N., Thompson A., Leach R.K. (2017). Feature-based characterisation of signature topography in laser powder bed fusion of metals. Meas. Sci. Technol..

[26] Thompson A., Senin N., Giusca C., Leach R. (2017). Topography of selectively laser melted surfaces: A comparison of different measurement methods. CIRP Ann..

[27] Newton L., Senin N., Chatzivagiannis E., Smith B., Leach R. (2020). Feature-based characterisation of Ti6Al4V electron beam powder bed fusion surfaces fabricated at different surface orientations. Addit. Manuf..

[28] Brown C.A., Hansen H.N., Jiang X.J., Blateyron F., Berglund J., Senin N., Bartkowiak T., Dixon B., Le Goïc G., Quinsat Y. (2018). Multiscale analyses and characterizations of surface topographies. CIRP Ann..

[29] Bartkowiak T., Berglund J., Brown C.A. (2020). Multiscale Characterizations of Surface Anisotropies. Materials.

[30] Quinsat Y., Lartigue C., Brown C.A., Hattali L. (2017). Characterization of surface topography of 3D printed parts by multi-scale analysis. Int. J. Interact. Des. Manuf..

[31] Quinsat Y., Lartigue C., Brown C.A., Hattali L. (2017). Multi-scale surface characterization in additive manufacturing using CT. Advances on Mechanics, Design Engineering and Manufacturing.

[32] GE Additive, 316L. https://www.ge.com/additive/sites/default/files/2019-11/316L-M2beide.pdf.

[33] Maleki I., Wolski M., Woloszynski T., Podsiadlo P., Stachowiak G. (2019). A Comparison of Multiscale Surface Curvature Characterization Methods for Tribological Surfaces. Tribol. Online.

[34] Bartkowiak T., Mendak M., Mrozek K., Wieczorowski M. (2020). Analysis of Surface Microgeometry Created by Electric Discharge Machining. Materials.

[35] Bartkowiak T., Berglund J., Brown C.A. (2018). Establishing functional correlations between multiscale areal curvatures and coefficients of friction for machined surfaces. Surf. Topogr. Metrol. Prop..

[36] Wang X., Shi T., Liao G., Zhang Y., Hong Y., Chen K. (2017). Using Wavelet Packet Transform for Surface Roughness Evaluation and Texture Extraction. Sensors.

[37] Zawada-Tomkiewicz A., Ściegienka R., Zuperl U., Stępień K. (2019). Images of the Machined Surface in Evaluation of the Efficiency of a Micro-Smoothing Process. Strojniški Vestn. J. Mech. Eng..

[38] Jiang X., Scott P., Whitehouse D. (2008). Wavelets and their applications for surface metrology. CIRP Ann..

[39] Gogolewski D., Makieła W., Nowakowski Ł. (2020). An assessment of applicability of the two-dimensionalwavelet transform to assess the minimum chip thickness determination accuracy. Metrol. Meas. Syst..

[40] Dutta S., Pal S.K., Sen R. (2016). Progressive tool flank wear monitoring by applying discrete wavelet transform on turned surface images. Measurement.

[41] Makieła W., Świderski J., Gogolewski D., Makieła K. Compensation of temperature errors when measuring surface textures by applying a two-dimensional wavelet transform. Proceedings of the 24th International Conference ENGINEERING MECHANICS 2018.

[42] Herrmann F.J. (1997). A Scaling Medium Representation a Discussion on Well-Logs, Fractals and Waves.

[43] Gogolewski D. (2020). Influence of the edge effect on the wavelet analysis process. Measurement.

[44] Gogolewski D. (2021). Fractional spline wavelets within the surface texture analysis. Measurement.

[45] Grzesik W., Brol S. (2009). Wavelet and fractal approach to surface roughness characterization after finish turning of different workpiece materials. J. Mater. Process. Technol..

[46] Edjeou W., Cerezo V., Zahouani H., Salvatore F. (2020). Multiscale analyses of pavement texture during polishing. Surf. Topogr. Metrol. Prop..

[47] Bruzzone A., Montanaro J., Ferrando A., Lonardo P. (2004). Wavelet Analysis for Surface Characterisation: An Experimental Assessment. CIRP Ann..

[48] Lee W., Ratnam M., Ahmad Z. (2017). Detection of chipping in ceramic cutting inserts from workpiece profile during turning using fast Fourier transform (FFT) and continuous wavelet transform (CWT). Precis. Eng..

[49] Chandra N.H., Sekhar A. (2016). Fault detection in rotor bearing systems using time frequency techniques. Mech. Syst. Signal Process..

[50] Zahouani H., Mezghani S., Vargiolu R., Dursapt M. (2008). Identification of manufacturing signature by 2D wavelet decomposition. Wear.

[51] Grzesik W., Żak K., Chudy R., Prażmowski M., Małecka J. (2019). Optimization of subtractive-transformative hybrid processes supported by the technological heredity concept. CIRP Ann..

[52] Lewandowski A., Feldshtein E. (2014). Technological heredity when roller burnishing of ductile cast iron. J. Sci. Ind. Res..

[53] Zmarzły P. (2020). Technological Heredity of the Turning Process. Teh. Vjesn. Tech. Gaz..

[54] Zmarzły P. (2020). Influence of bearing raceway surface topography on the level of generated vibration as an example of operational heredity. Ind. J. Eng. Mater. Sci..

[55] Newton L., Senin N., Smith B., Leach R. Feature-based characterisation of evolving surface topographies in finishing operations for additive manufacturing. Proceedings of the European Society for Precision Engineering and Nanotechnology, Conference Proceedings—18th International Conference and Exhibition, EUSPEN 2018.

[56] Todhunter L., Leach R., Lawes S., Blateyron F. (2017). Industrial survey of ISO surface texture parameters. CIRP J. Manuf. Sci. Technol..

[57] Leach R., Bourell D., Carmignato S., Donmez A., Senin N., Dewulf W. (2019). Geometrical metrology for metal additive manufacturing. CIRP Ann..

