# Supplementary material for: Multiscale Analysis of Surface Texture Quality of Models Manufactured by Laser Powder-Bed Fusion Technology and Machining from 316L Steel
Source: Materials (Basel). 2021 May 24;14(11):2794. doi: 10.3390/ma14112794 (PMC8197311; doi:10.3390/ma14112794)
Supplement: Supplementary file 1 [file materials-14-02794-s001.zip › materials-1237036-supplementary - for publish.pdf]

materials

MDPI

Supplementary Materials

Multiscale Analysis of Surface Texture Quality of Models Manufactured by Laser Powder-Bed Fusion Technology and Machining from 316L Steel

Damian Gogolewski [1,*], Tomasz Bartkowiak [2], Tomasz Kozior [1] and Paweł Zmarzły [1]

[1] Department of Manufacturing Technology and Metrology, Kielce University of Technology, 25-314 Kielce, Poland; tkozior@tu.kielce.pl (T.K.); pzmarzly@tu.kielce.pl (P.Z.)
[2] Institute of Mechanical Technology, Poznan University of Technology; 60-965 Poznań, Poland; tomasz.bartkowiak@put.poznan.pl
* Correspondence: dgogolewski@tu.kielce.pl

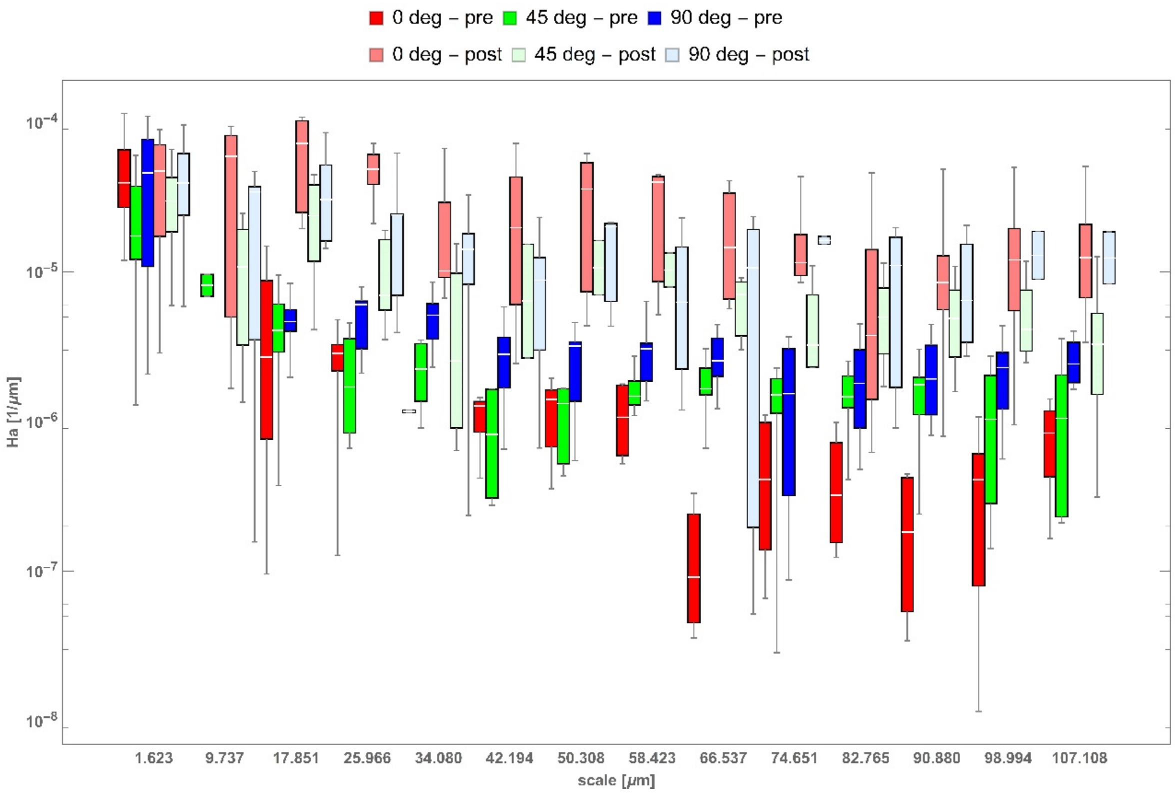

Figure S1. Evolution of selected curvature parameters with scale—average mean curvature—Ha. Please note that “pre” and “post” terms refer to post-processing via machining.

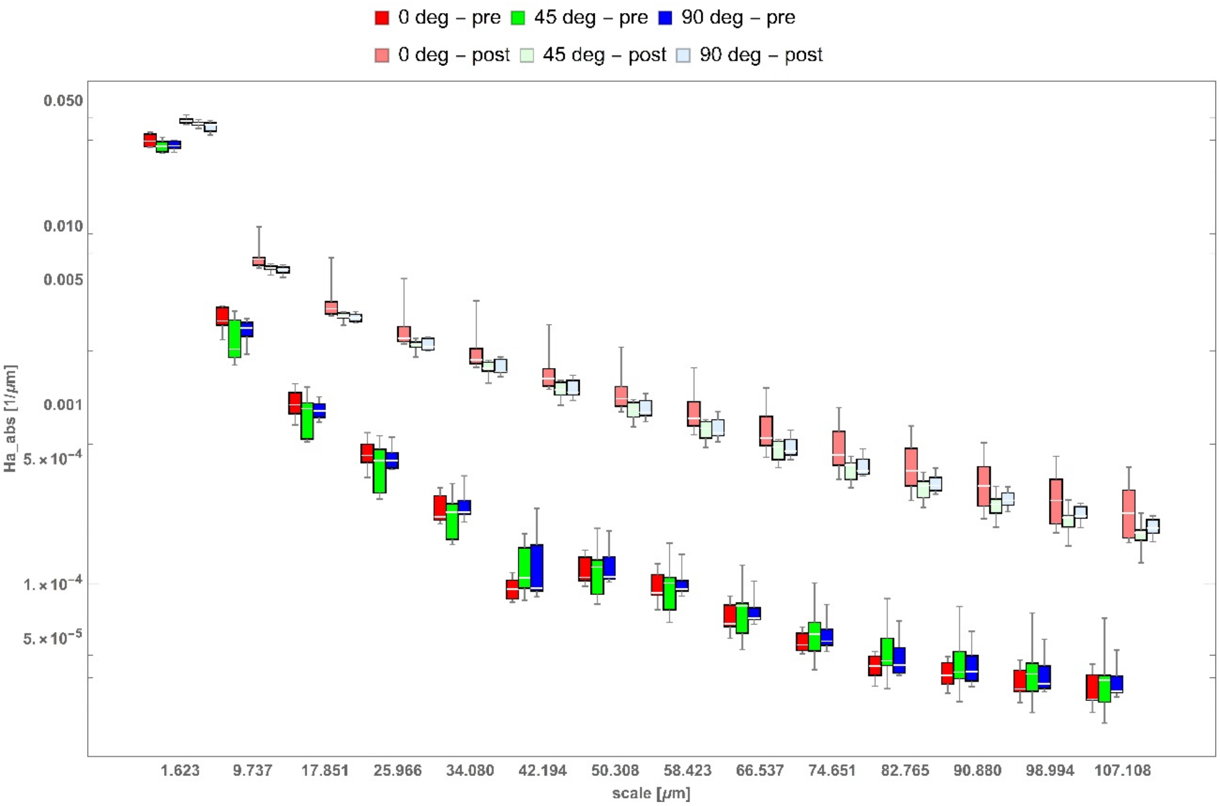

Figure S2. Evolution of selected curvature parameters with scale—average absolute mean curvature—Ha_{abs}, Please note that "pre" and "post" terms refer to post-processing via machining.

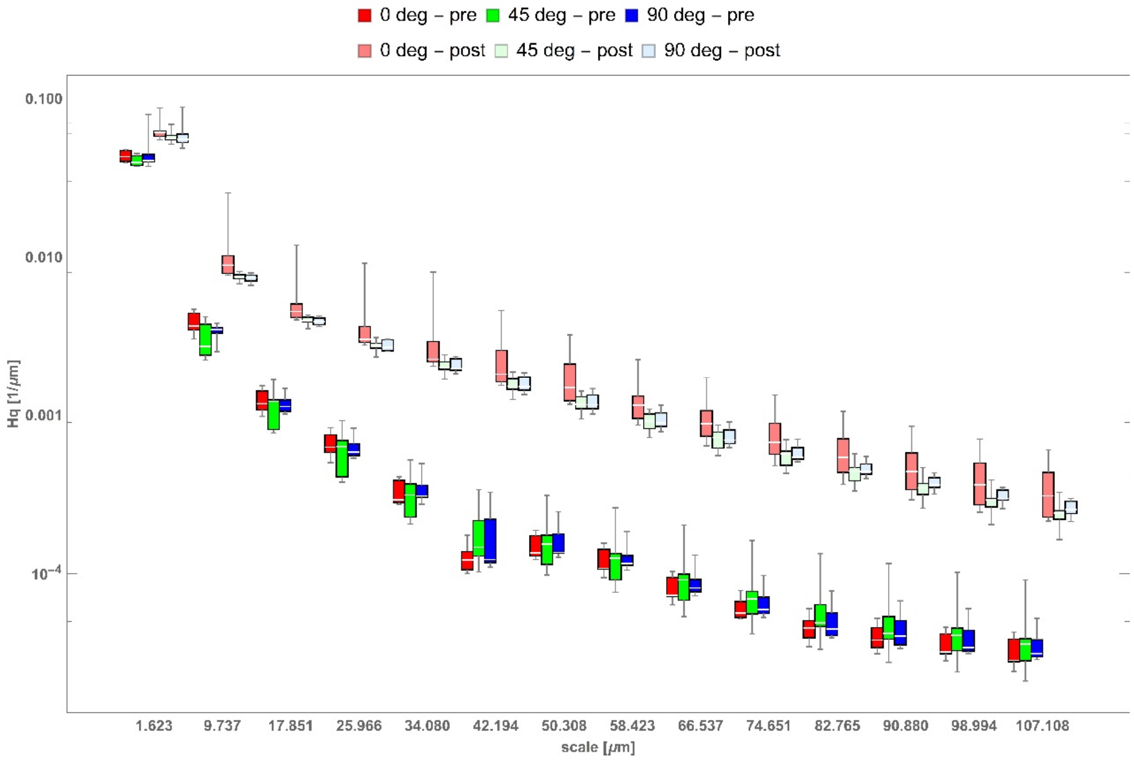

Figure S3. Evolution of selected curvature parameters with scale—SD of mean curvature—Hq. Please note that "pre" and "post" terms refer to post-processing via machining.

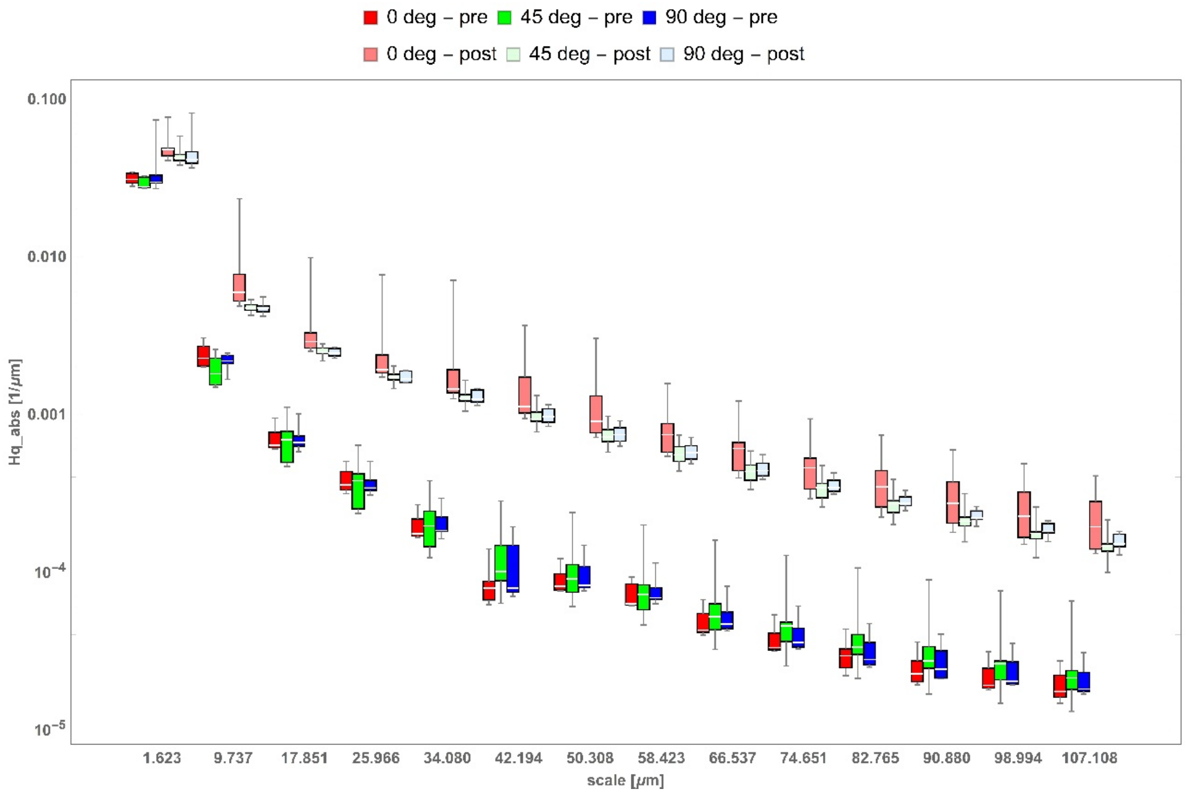

Figure S4. Evolution of selected curvature parameters with scale—SD of absolute mean curvature—Hq_{abs}, Please note that "pre" and "post" terms refer to post-processing via machining.

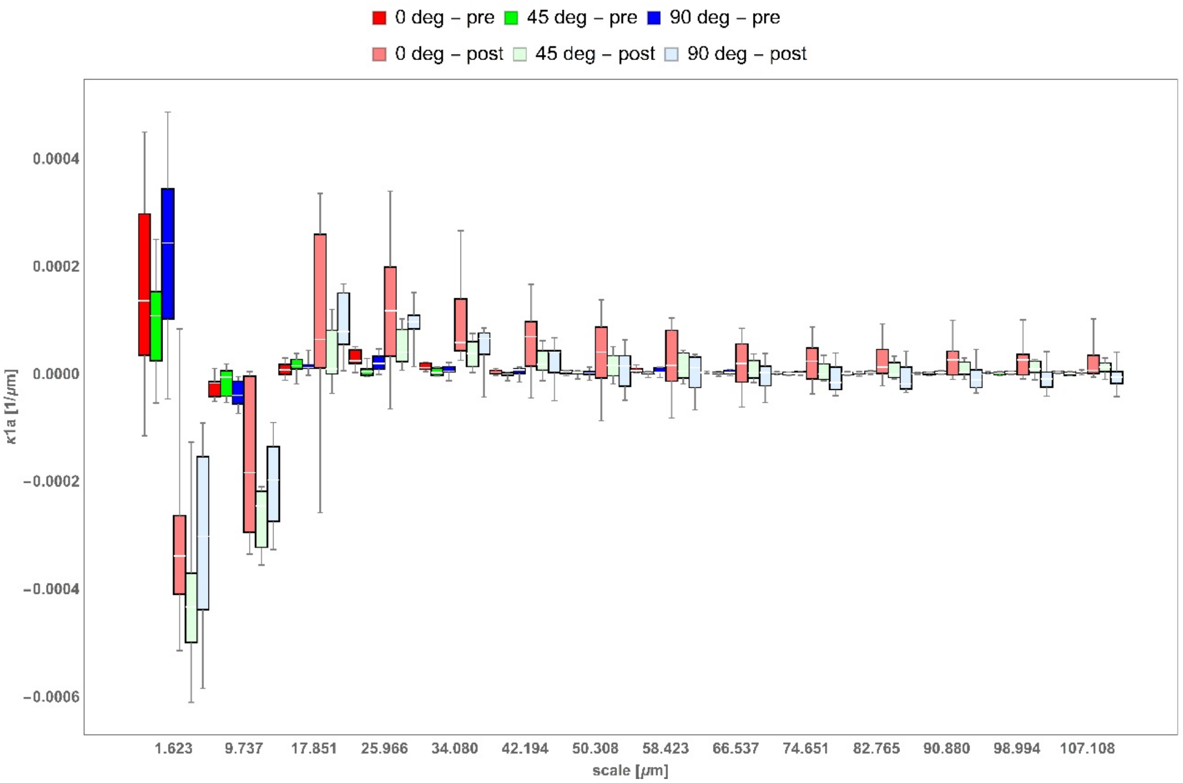

Figure S5. Evolution of selected curvature parameters with scale—average maximum curvature—$\kappa 1a$. Please note that "pre" and "post" terms refer to post-processing via machining.

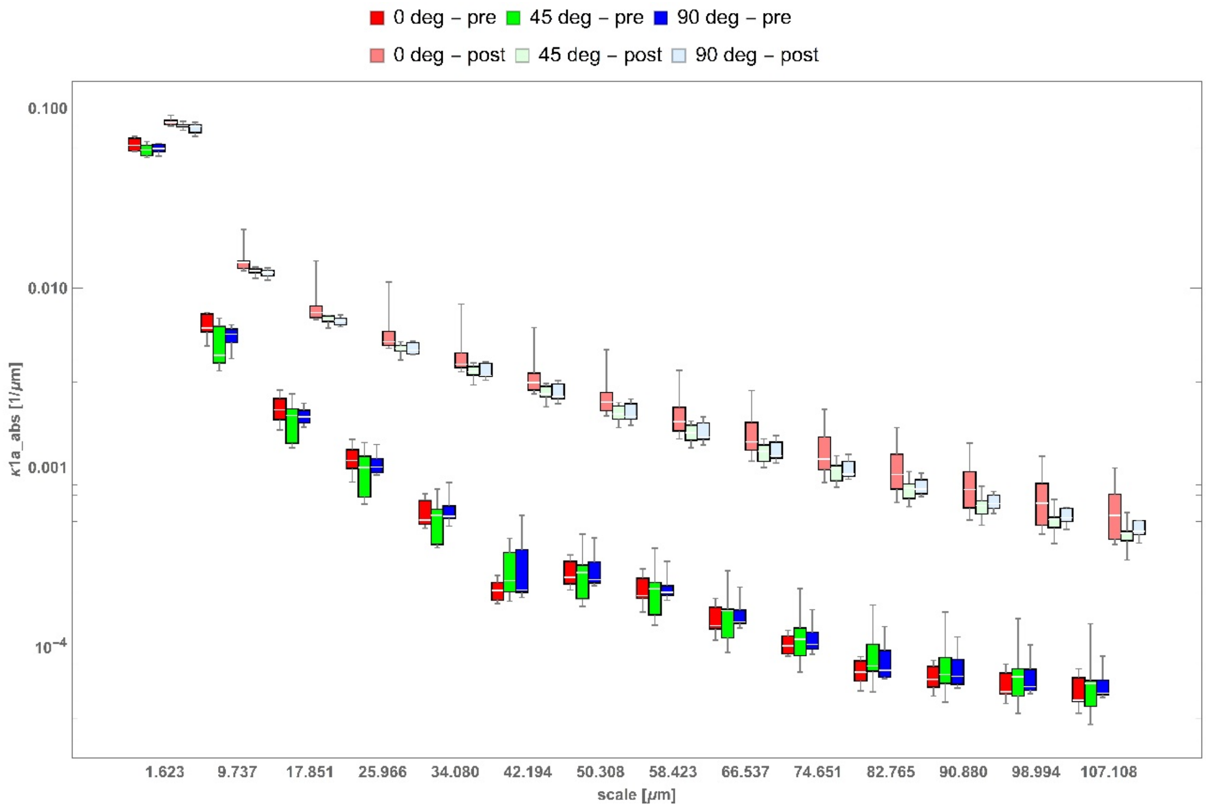

Figure S6. Evolution of selected curvature parameters with scale—average absolute maximum curvature—$\kappa 1a_{abs}$. Please note that "pre" and "post" terms refer to post-processing via machining.

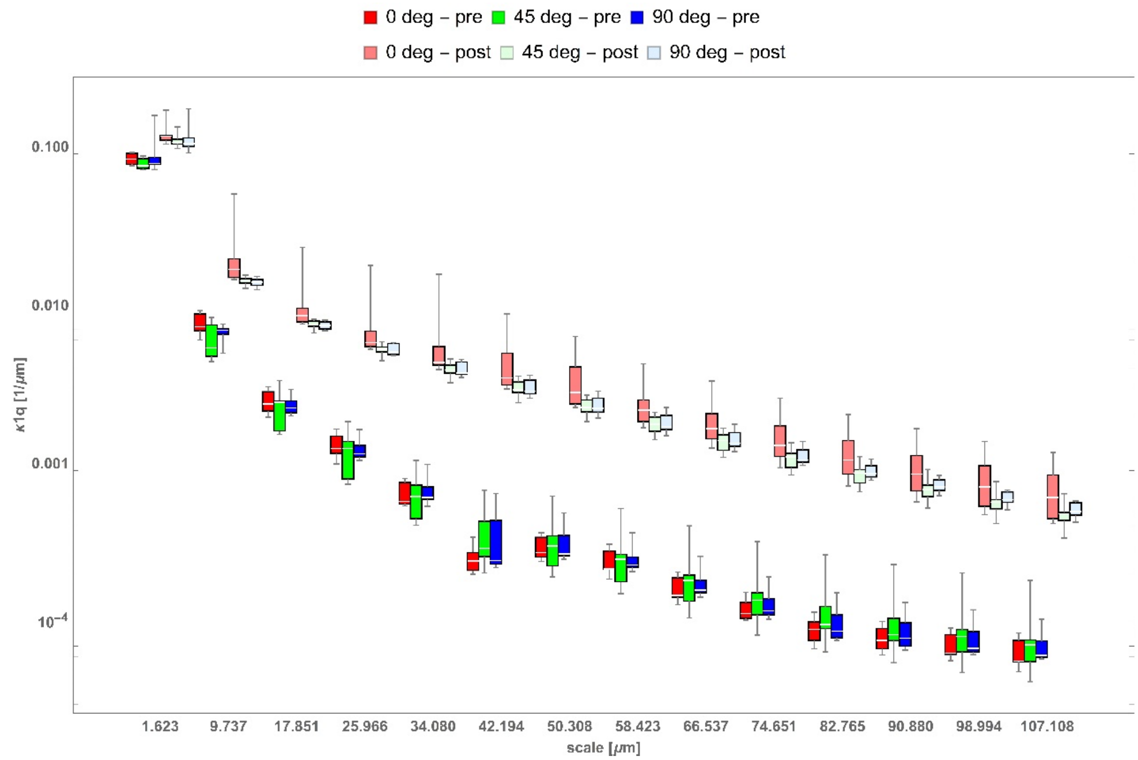

Figure S7. Evolution of selected curvature parameters with scale—SD of maximum curvature—$\kappa 1q$. Please note that "pre" and "post" terms refer to post-processing via machining.

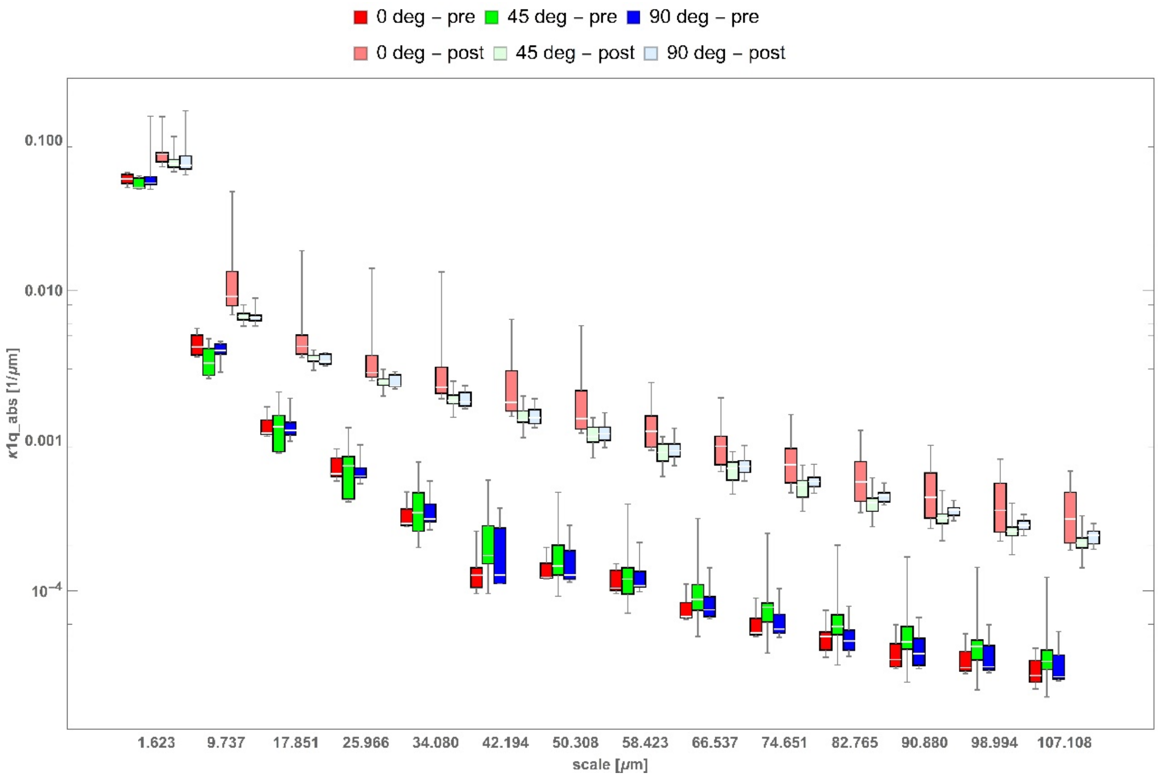

Figure S8. Evolution of selected curvature parameters with scale—SD of absolute maximum curvature—$\kappa 1q_{abs}$. Please note that "pre" and "post" terms refer to post-processing via machining.

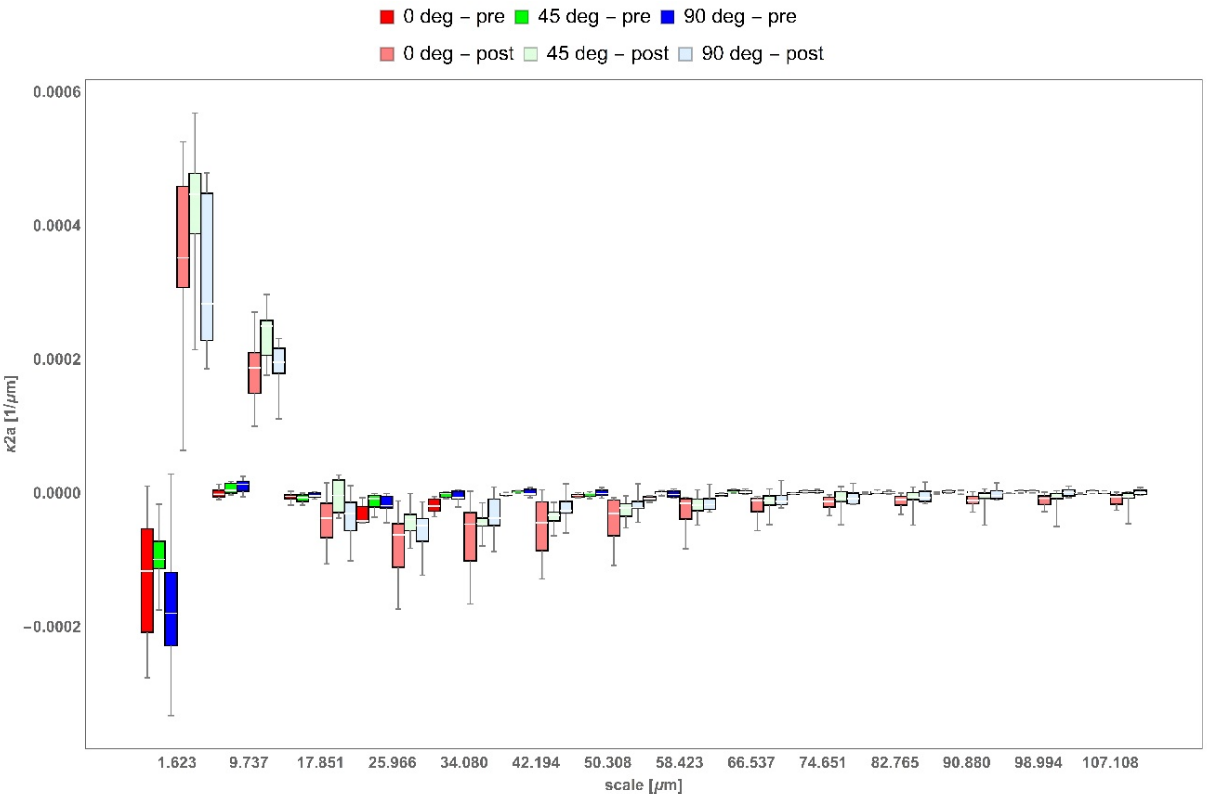

Figure S9. Evolution of selected curvature parameters with scale—average minimum curvature—$\kappa 2a$. Please note that "pre" and "post" terms refer to post-processing via machining.

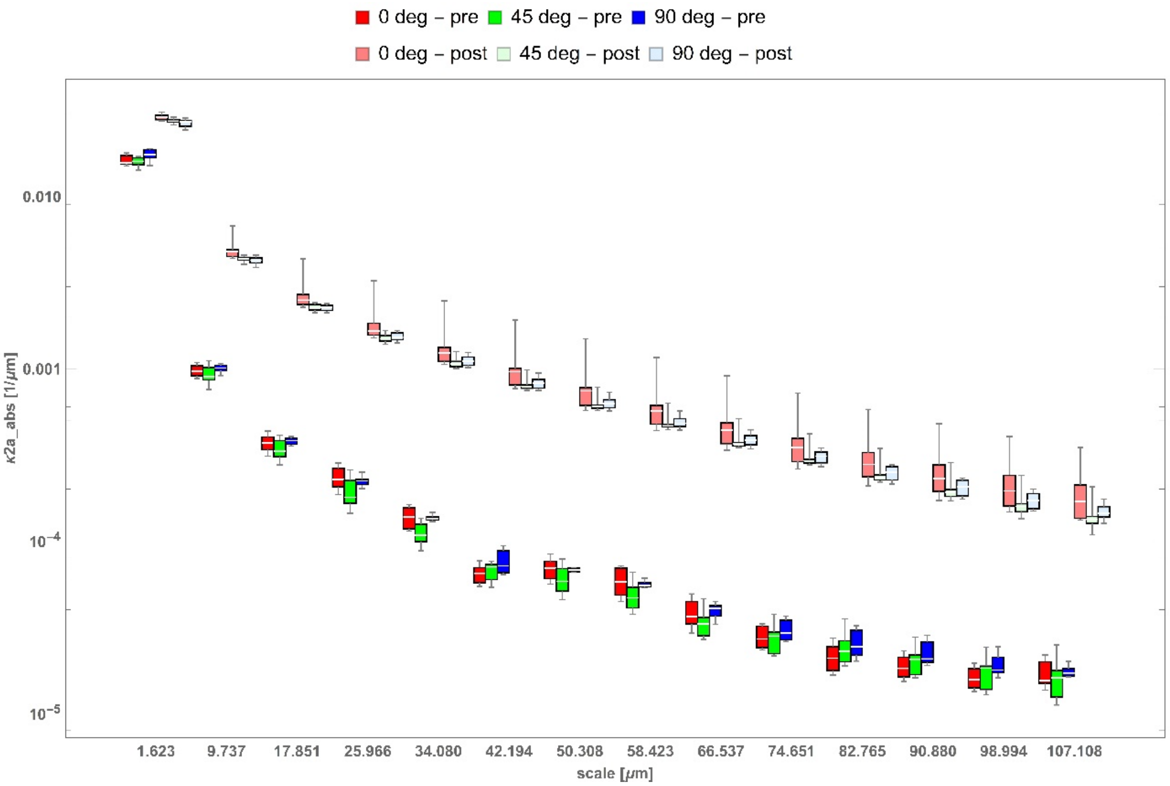

Figure S10. Evolution of selected curvature parameters with scale—average absolute minimum curvature—$\kappa 2a_{abs}$. Please note that "pre" and "post" terms refer to post-processing via machining.

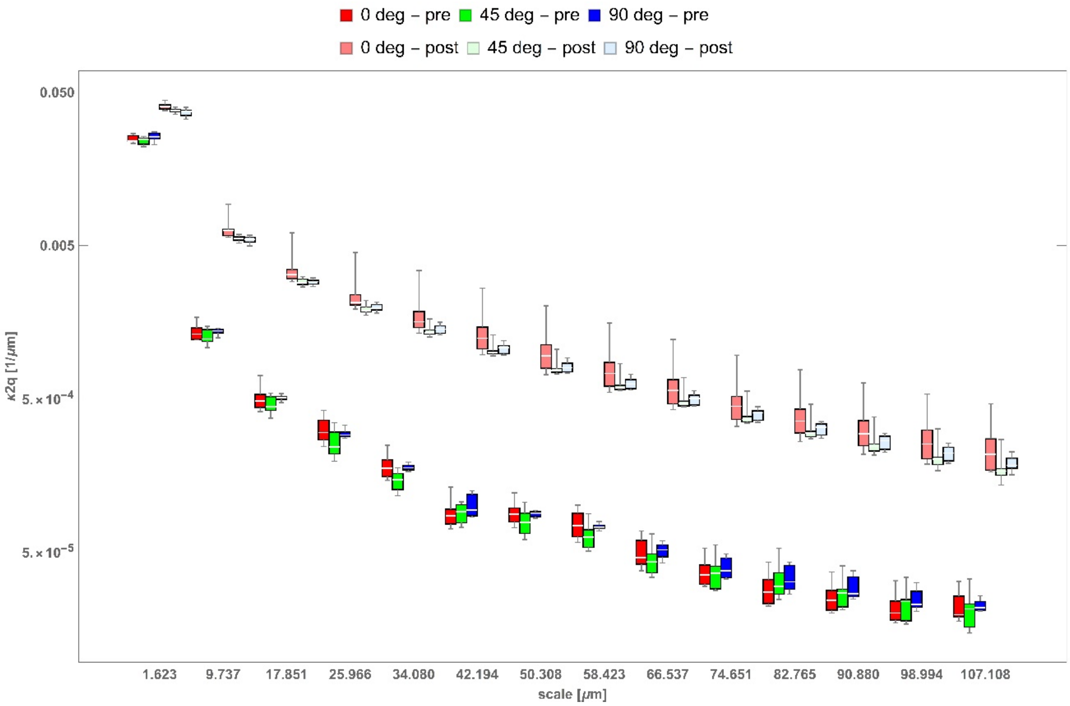

Figure S11. Evolution of selected curvature parameters with scale—SD of minimum curvature—κ2q. Please note that "pre" and "post" terms refer to post-processing via machining.

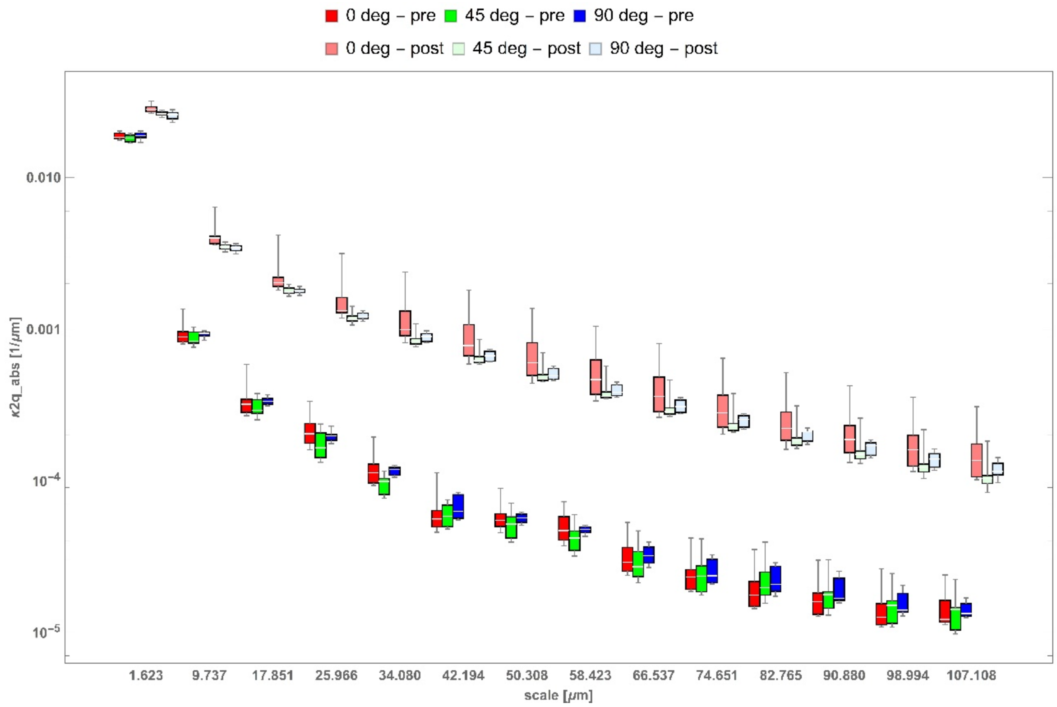

Figure S12. Evolution of selected curvature parameters with scale—SD of absolute minimum curvature—$\kappa 2q_{abs}$. Please note that "pre" and "post" terms refer to post-processing via machining.

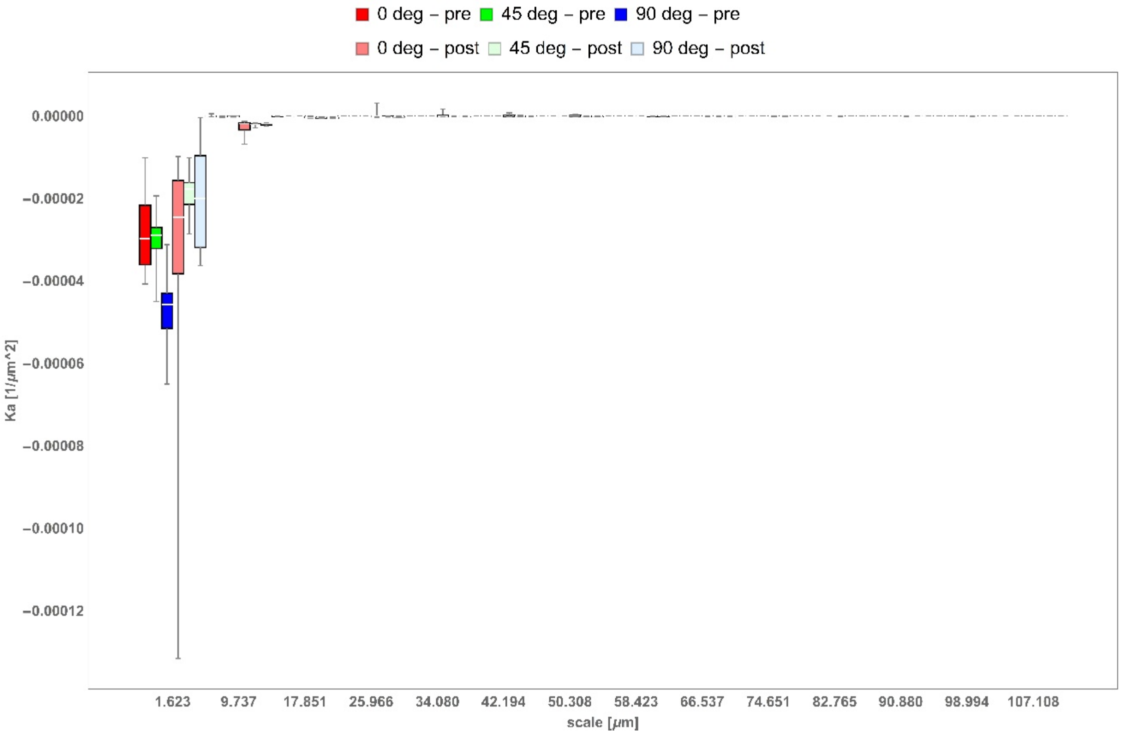

Figure S13. Evolution of selected curvature parameters with scale—average Gaussian curvature—Ka. Please note that "pre" and "post" terms refer to post-processing via machining.

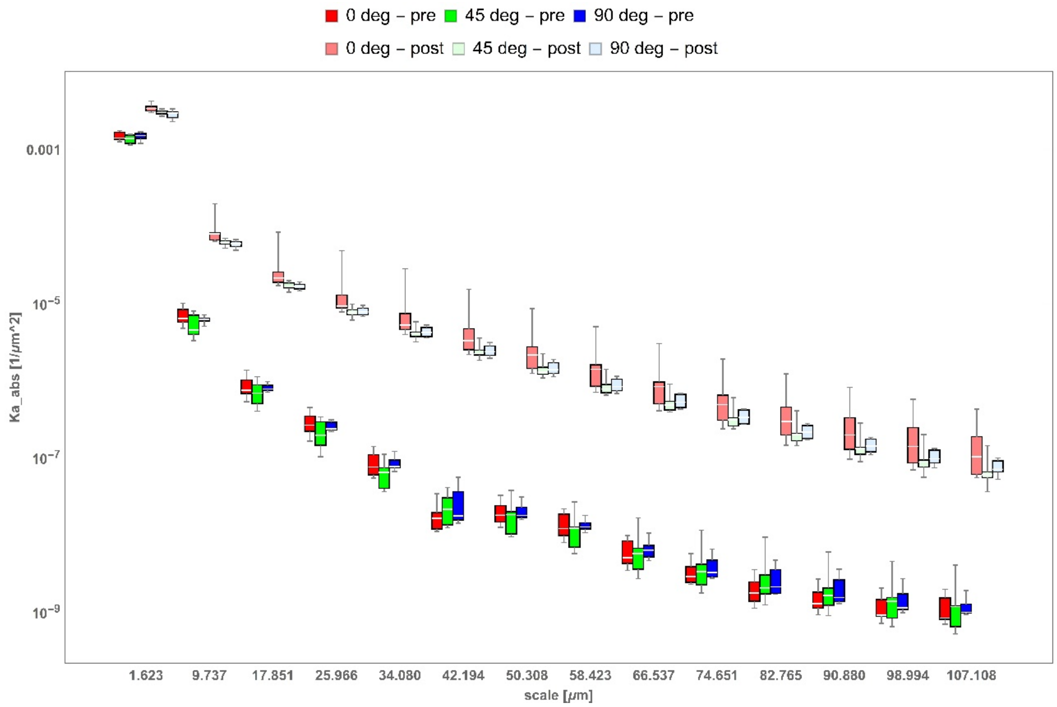

Figure S14. Evolution of selected curvature parameters with scale—average absolute Gaussian curvature—Ka_{abs}. Please note that "pre" and "post" terms refer to post-processing via machining.

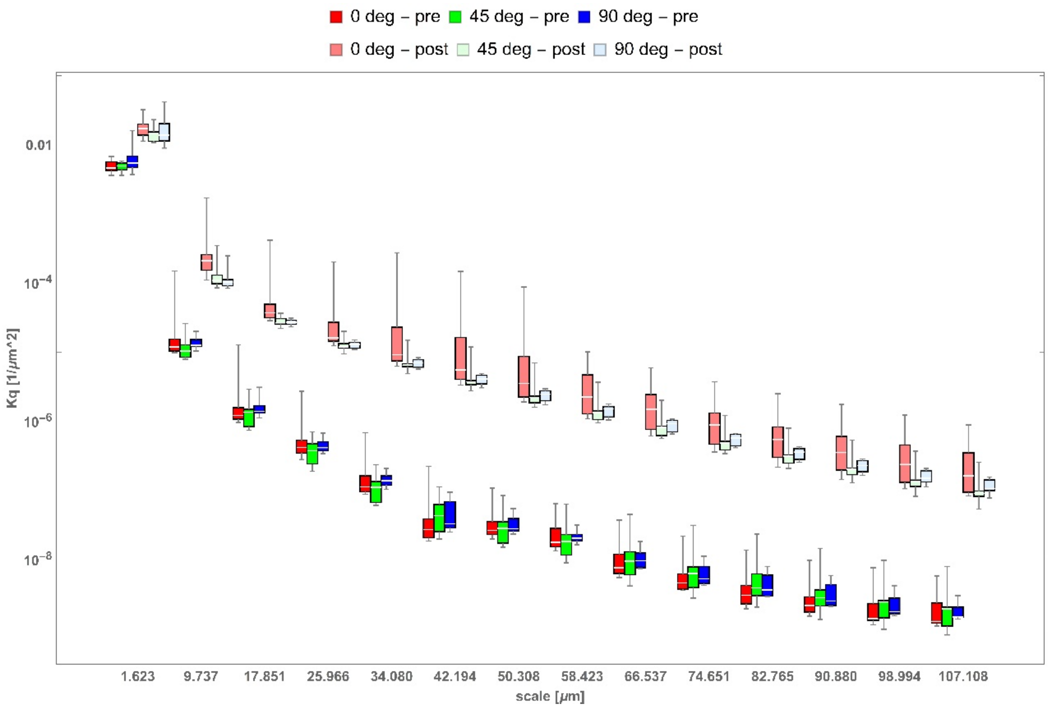

Figure S15. Evolution of selected curvature parameters with scale—SD of Gaussian curvature—Kq. Please note that "pre" and "post" terms refer to post-processing via machining.

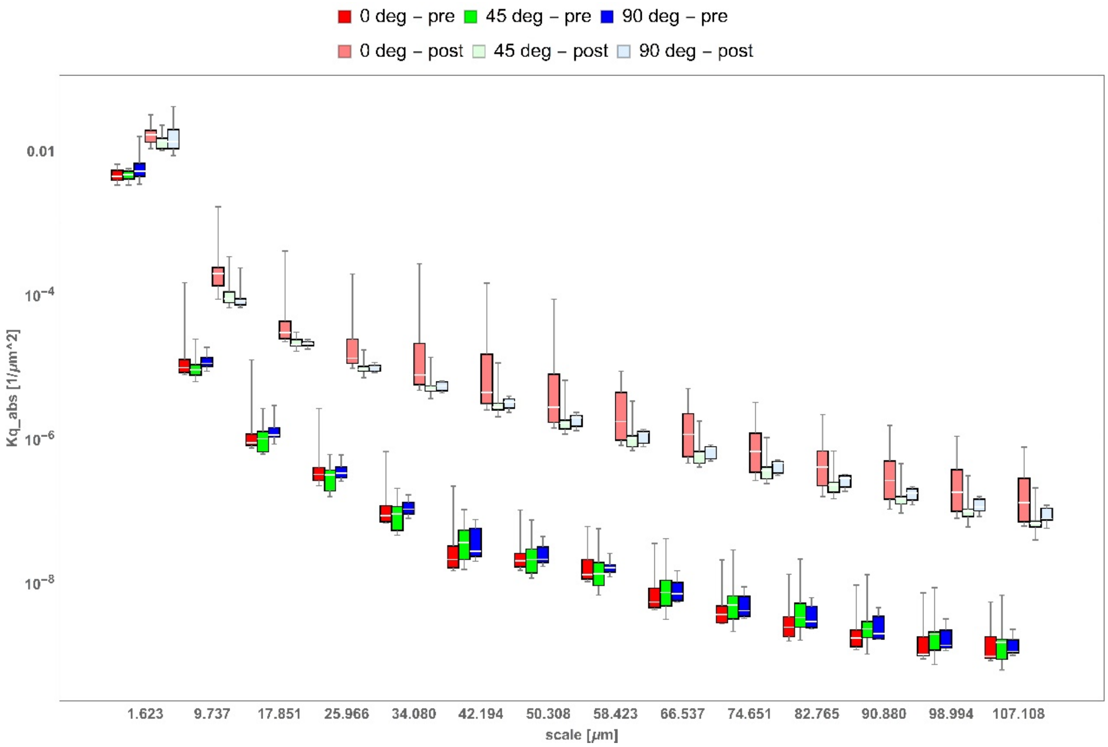

Figure S16. Evolution of selected curvature parameters with scale—SD of absolute Gaussian curvature—Kq_{abs}. Please note that "pre" and "post" terms refer to post-processing via machining.

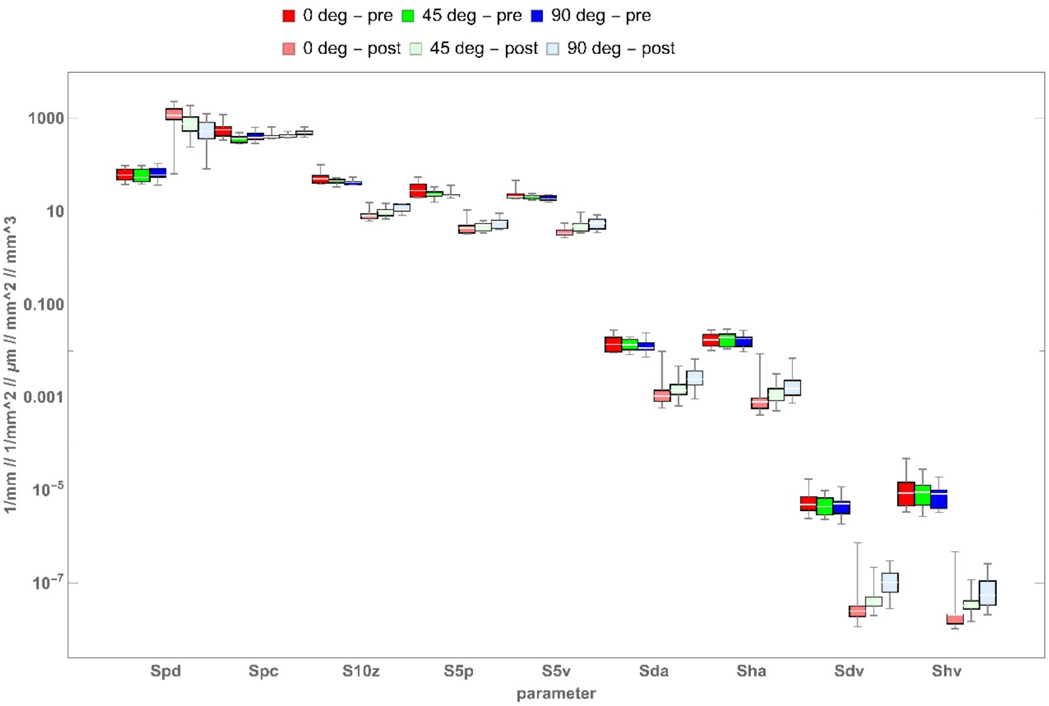

Figure S17. Feature areal parameters.

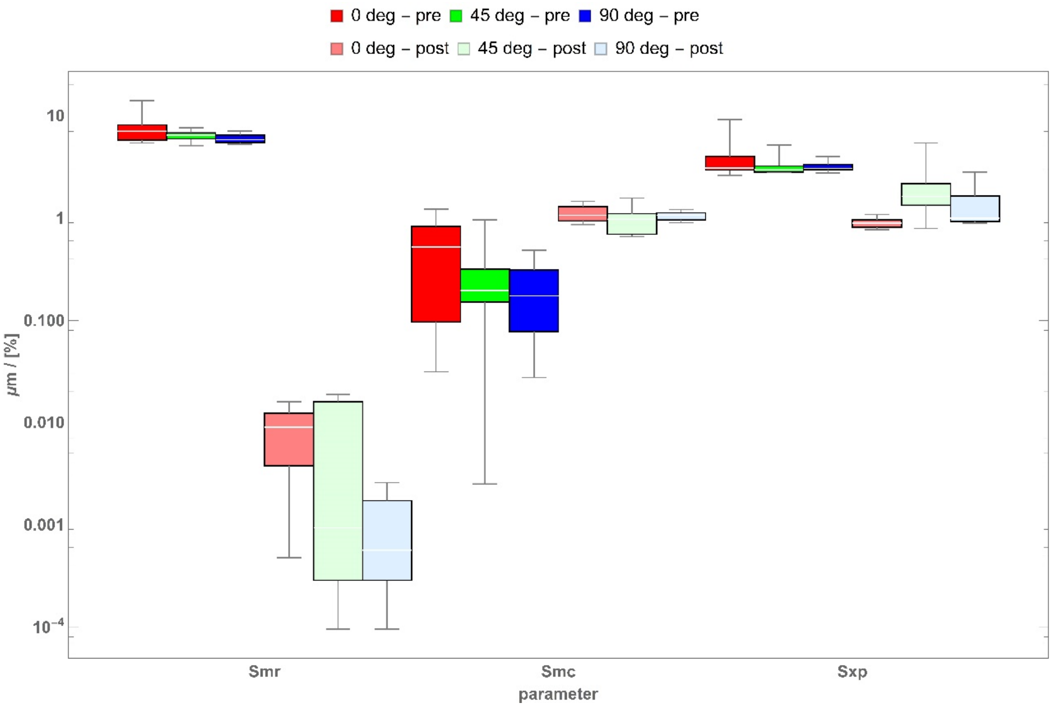

Figure S18. Functional areal parameters—part 1.

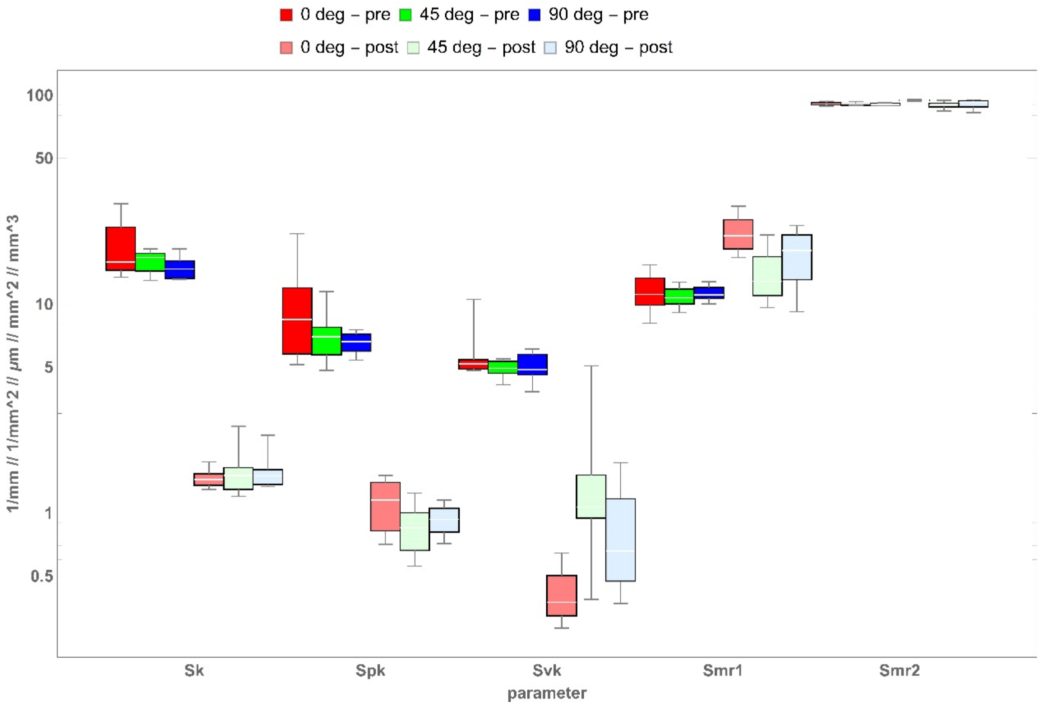

Figure S19. Functional areal parameters—part 2.

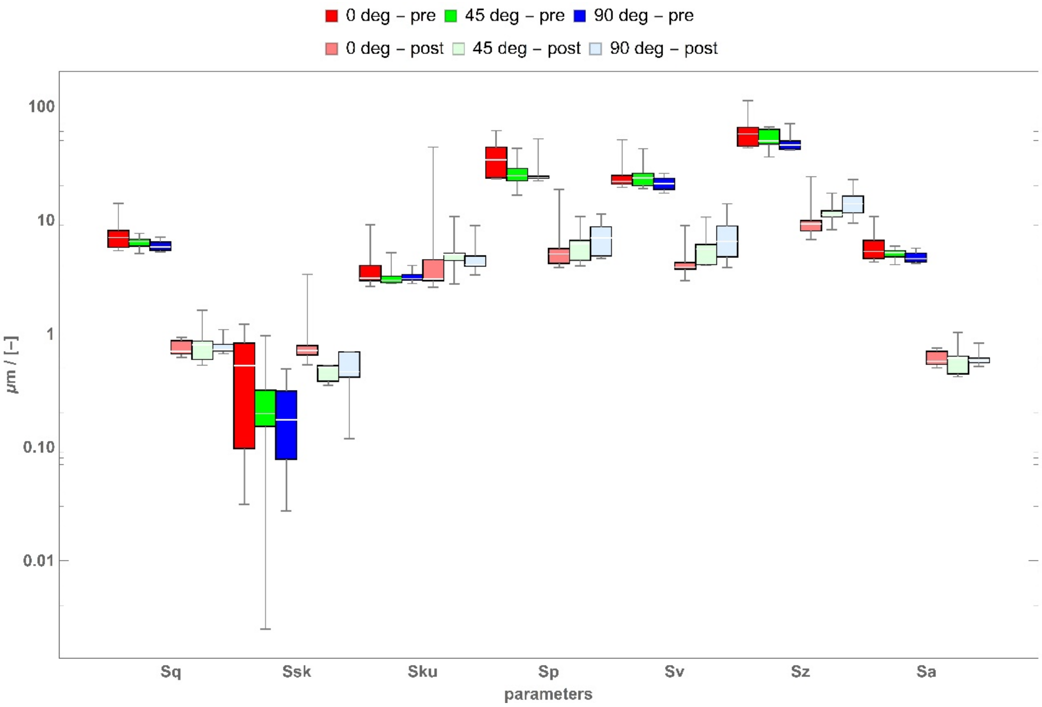

Figure S20. Height areal parameters.

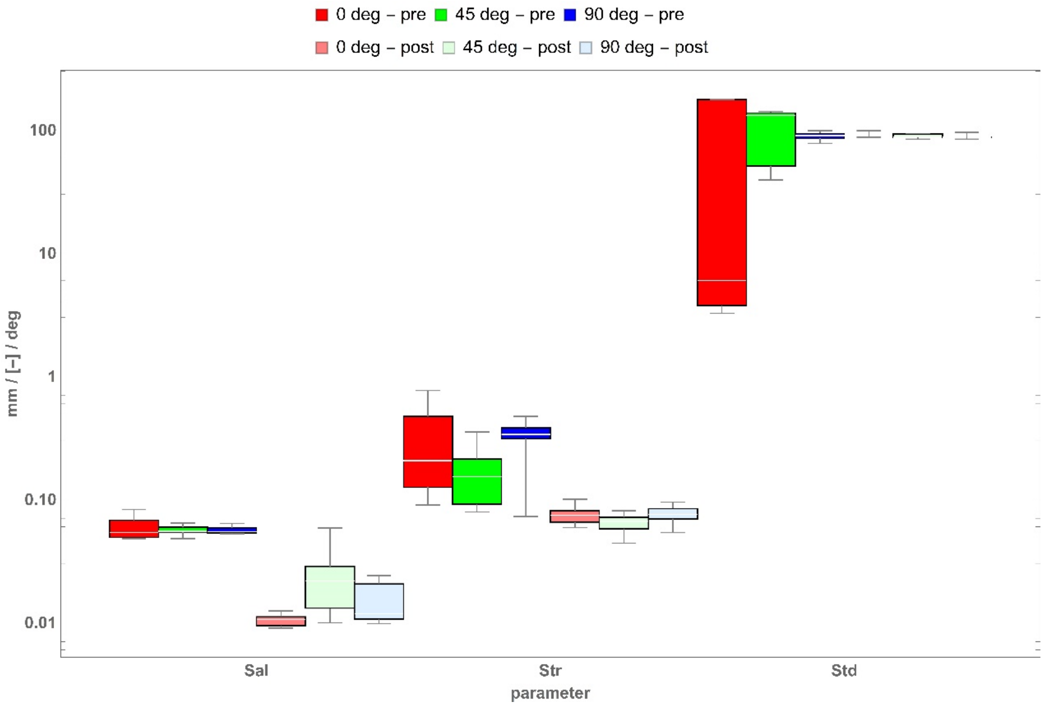

Figure S21. Spatial areal parameters.

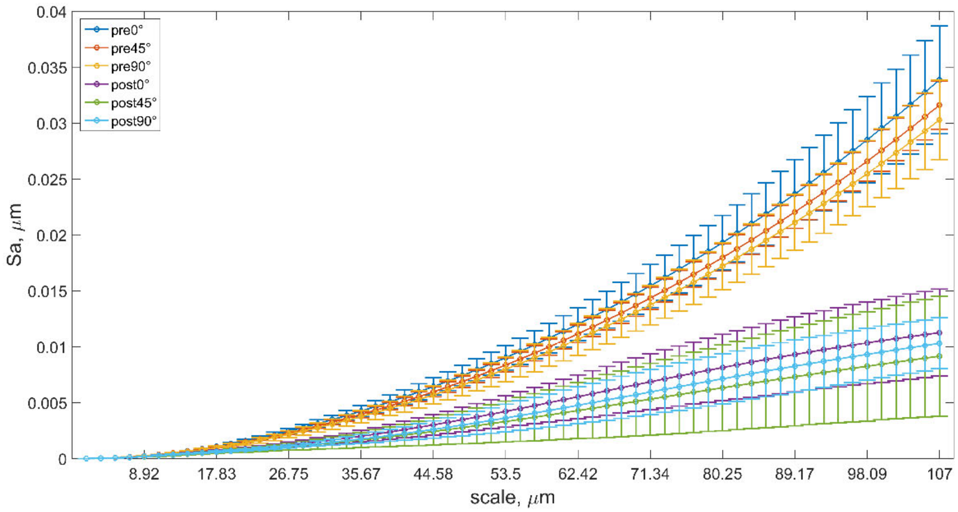

Figure S22. Evolution of arithmetical mean height (Sa) with scale. Please note that "pre" and "post" terms refer to post-processing via machining.

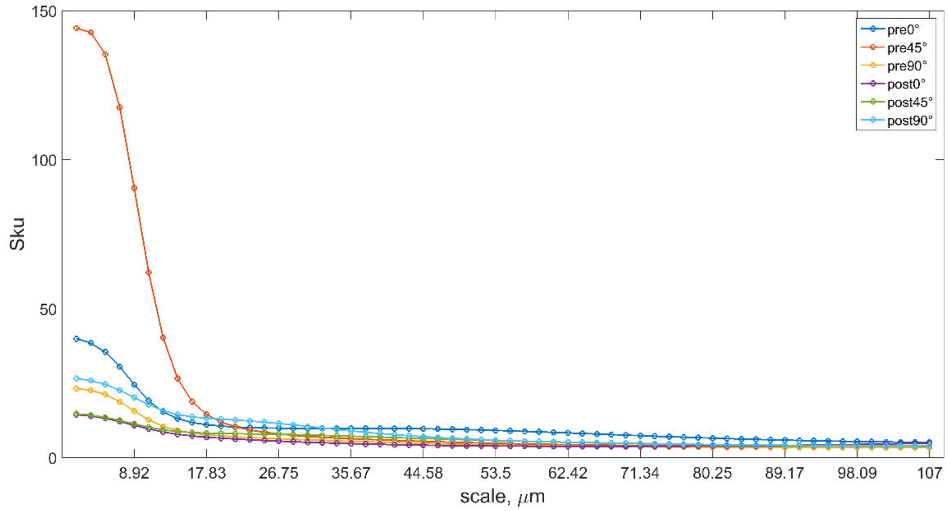

Figure S23. Evolution of kurtosis (Sku) with scale. Please note that "pre" and "post" terms refer to post-processing via machining.

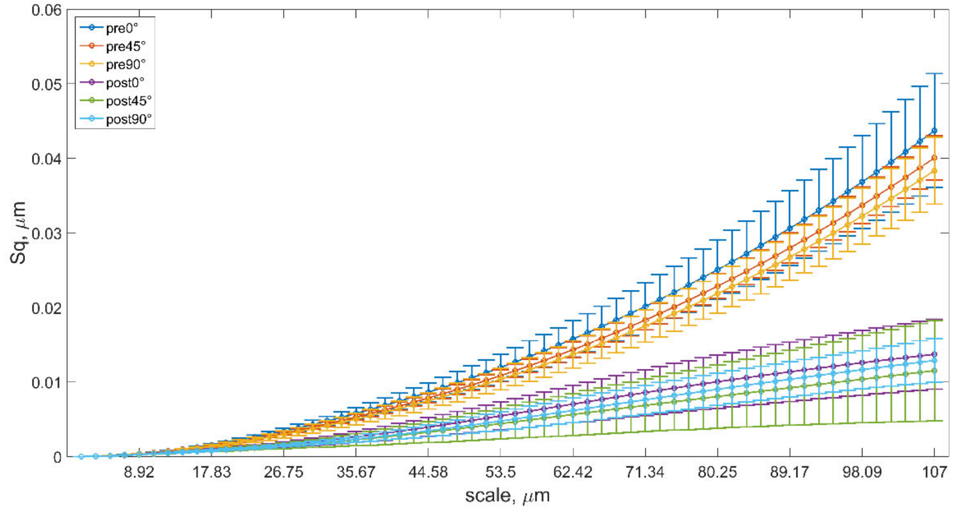

Figure S24. Evolution of root mean square height (Sq) with scale. Please note that "pre" and "post" terms refer to post-processing via machining.

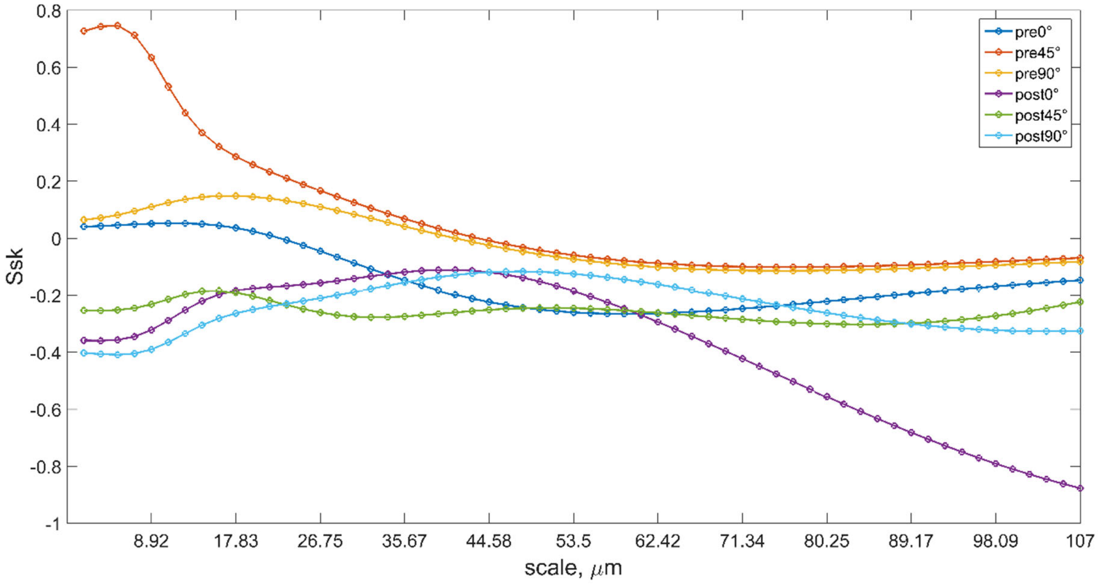

Figure S25. Evolution of skewness (Ssk) with scale. Please note that "pre" and "post" terms refer to post-processing via machining.

scale, µm	κ1a	κ1q	κ2a	κ2q	Ha	Hq	Ka	Kq	κ1a_abs	κ1q_abs	κ2a_abs	κ2q_abs	Ha_abs	Hq_abs	Ka_abs	Kq_abs
1.623	0.020	0.354	0.012	0.002	0.250	0.337	0.101	0.388	0.000	0.456	0.031	0.000	0.000	0.429	0.000	0.393
4.869	0.442	0.000	0.432	0.000	0.640	0.000	0.719	0.004	0.000	0.000	0.000	0.000	0.000	0.000	0.000	0.005
8.114	0.290	0.001	0.111	0.000	0.366	0.001	0.416	0.068	0.000	0.006	0.000	0.000	0.000	0.004	0.002	0.073
9.737	0.260	0.013	0.032	0.001	0.549	0.012	0.346	0.128	0.000	0.029	0.000	0.001	0.000	0.028	0.008	0.130
11.360	0.183	0.004	0.024	0.002	0.458	0.003	0.315	0.115	0.000	0.013	0.001	0.003	0.000	0.011	0.018	0.120
14.606	0.431	0.022	0.006	0.006	0.821	0.020	0.354	0.169	0.003	0.056	0.005	0.008	0.003	0.047	0.045	0.179
17.851	0.473	0.048	0.038	0.010	0.810	0.044	0.309	0.189	0.009	0.105	0.010	0.011	0.009	0.089	0.067	0.199
17.851	0.473	0.048	0.038	0.010	0.810	0.044	0.309	0.189	0.009	0.105	0.010	0.011	0.009	0.089	0.067	0.199
21.097	0.242	0.062	0.007	0.012	0.708	0.056	0.219	0.139	0.018	0.131	0.011	0.012	0.016	0.107	0.080	0.147
24.343	0.067	0.110	0.014	0.013	0.440	0.102	0.265	0.251	0.024	0.175	0.013	0.013	0.022	0.155	0.092	0.258
25.966	0.069	0.070	0.014	0.014	0.445	0.065	0.253	0.175	0.025	0.118	0.015	0.014	0.022	0.103	0.095	0.182
27.588	0.069	0.077	0.016	0.016	0.491	0.071	0.246	0.169	0.026	0.123	0.018	0.014	0.022	0.110	0.098	0.175
30.834	0.057	0.085	0.016	0.020	0.533	0.077	0.196	0.144	0.031	0.129	0.025	0.015	0.026	0.116	0.100	0.148
34.080	0.058	0.104	0.030	0.023	0.411	0.092	0.100	0.181	0.037	0.146	0.034	0.016	0.031	0.129	0.096	0.183
42.194	0.131	0.033	0.100	0.041	0.675	0.031	0.062	0.129	0.057	0.037	0.076	0.028	0.047	0.040	0.067	0.134
50.308	0.335	0.016	0.107	0.047	0.932	0.014	0.042	0.198	0.030	0.046	0.071	0.035	0.023	0.038	0.067	0.213
58.423	0.366	0.020	0.072	0.064	0.971	0.019	0.085	0.042	0.028	0.011	0.084	0.053	0.020	0.025	0.082	0.044
66.537	0.320	0.023	0.133	0.094	0.884	0.022	0.903	0.052	0.027	0.016	0.119	0.081	0.018	0.035	0.089	0.050
74.651	0.104	0.024	0.225	0.113	0.387	0.023	0.771	0.064	0.028	0.015	0.140	0.098	0.018	0.037	0.091	0.061
82.765	0.071	0.030	0.320	0.126	0.227	0.030	0.471	0.074	0.037	0.018	0.155	0.109	0.026	0.040	0.087	0.072
90.880	0.043	0.039	0.185	0.098	0.211	0.040	0.328	0.078	0.044	0.027	0.115	0.091	0.037	0.043	0.077	0.079
98.994	0.034	0.045	0.064	0.076	0.213	0.045	0.316	0.075	0.051	0.031	0.088	0.074	0.047	0.041	0.067	0.078
107.108	0.062	0.038	0.068	0.046	0.312	0.038	0.282	0.064	0.042	0.030	0.051	0.047	0.040	0.034	0.055	0.068

Figure S26. ANOVA—Curvature parameters—factor 1—build direct.

scale, µm	κ1a	κ1q	κ2a	κ2q	Ha	Hq	Ka	Kq	κ1a_abs	κ1q_abs	κ2a_abs	κ2q_abs	Ha_abs	Hq_abs	Ka_abs	Kq_abs
1.623	0.000	0.000	0.000	0.000	0.248	0.000	0.018	0.000	0.000	0.000	0.000	0.000	0.000	0.000	0.000	0.000
4.869	0.000	0.000	0.000	0.000	0.000	0.000	0.000	0.000	0.000	0.000	0.000	0.000	0.000	0.000	0.000	0.000
8.114	0.000	0.000	0.000	0.000	0.633	0.000	0.000	0.000	0.000	0.000	0.000	0.000	0.000	0.000	0.000	0.000
9.737	0.000	0.000	0.000	0.000	0.350	0.000	0.000	0.002	0.000	0.000	0.000	0.000	0.000	0.000	0.000	0.005
11.360	0.000	0.000	0.000	0.000	0.107	0.000	0.000	0.000	0.000	0.000	0.000	0.000	0.000	0.000	0.000	0.000
14.606	0.667	0.000	0.000	0.000	0.132	0.000	0.000	0.000	0.000	0.000	0.000	0.000	0.000	0.000	0.000	0.002
17.851	0.008	0.000	0.001	0.000	0.193	0.000	0.000	0.003	0.000	0.000	0.000	0.000	0.000	0.000	0.000	0.009
17.851	0.008	0.000	0.001	0.000	0.193	0.000	0.000	0.003	0.000	0.000	0.000	0.000	0.000	0.000	0.000	0.009
21.097	0.000	0.000	0.000	0.000	0.174	0.000	0.010	0.000	0.000	0.000	0.000	0.000	0.000	0.000	0.000	0.001
24.343	0.000	0.000	0.000	0.000	0.149	0.000	0.604	0.034	0.000	0.000	0.000	0.000	0.000	0.000	0.000	0.059
25.966	0.000	0.000	0.000	0.000	0.162	0.000	0.665	0.003	0.000	0.000	0.000	0.000	0.000	0.000	0.000	0.008
27.588	0.000	0.000	0.000	0.000	0.215	0.000	0.705	0.003	0.000	0.000	0.000	0.000	0.000	0.000	0.000	0.008
30.834	0.000	0.000	0.000	0.000	0.350	0.000	0.797	0.003	0.000	0.000	0.000	0.000	0.000	0.000	0.000	0.008
34.080	0.000	0.000	0.000	0.000	0.532	0.000	0.932	0.050	0.000	0.000	0.000	0.000	0.000	0.000	0.000	0.072
42.194	0.001	0.000	0.000	0.000	0.487	0.000	0.539	0.033	0.000	0.000	0.000	0.000	0.000	0.000	0.000	0.050
50.308	0.034	0.000	0.000	0.000	0.313	0.000	0.031	0.046	0.000	0.000	0.000	0.000	0.000	0.000	0.000	0.071
58.423	0.254	0.000	0.000	0.000	0.305	0.000	0.000	0.000	0.000	0.000	0.000	0.000	0.000	0.000	0.000	0.000
66.537	0.285	0.000	0.000	0.000	0.347	0.000	0.000	0.000	0.000	0.000	0.000	0.000	0.000	0.000	0.000	0.000
74.651	0.236	0.000	0.000	0.000	0.493	0.000	0.000	0.000	0.000	0.000	0.000	0.000	0.000	0.000	0.000	0.000
82.765	0.153	0.000	0.001	0.000	0.718	0.000	0.000	0.000	0.000	0.000	0.000	0.000	0.000	0.000	0.000	0.000
90.880	0.072	0.000	0.001	0.000	0.932	0.000	0.000	0.000	0.000	0.000	0.000	0.000	0.000	0.000	0.000	0.000
98.994	0.061	0.000	0.001	0.000	0.961	0.000	0.000	0.000	0.000	0.000	0.000	0.000	0.000	0.000	0.000	0.000
107.108	0.116	0.000	0.003	0.000	0.976	0.000	0.000	0.000	0.000	0.000	0.000	0.000	0.000	0.000	0.000	0.000

Figure S27. ANOVA—Curvature parameters—factor 2—machining.

scale, µm	κ1a	κ1q	κ2a	κ2q	Ha	Hq	Ka	Kq	κ1a_abs	κ1q_abs	κ2a_abs	κ2q_abs	Ha_abs	Hq_abs	Ka_abs	Kq_abs
1.622851	0.906	0.493	0.732	0.000	0.524	0.453	0.013	0.485	0.070	0.625	0.000	0.001	0.108	0.519	0.001	0.487
4.868553	0.454	0.050	0.444	0.000	0.719	0.045	0.439	0.004	0.202	0.019	0.000	0.000	0.274	0.015	0.002	0.004
8.114255	0.203	0.185	0.232	0.002	0.149	0.173	0.178	0.235	0.275	0.138	0.002	0.004	0.316	0.119	0.017	0.234
9.737106	0.168	0.178	0.100	0.006	0.382	0.173	0.221	0.242	0.248	0.116	0.004	0.010	0.283	0.120	0.033	0.241
11.35996	0.171	0.139	0.018	0.013	0.710	0.130	0.315	0.205	0.206	0.074	0.008	0.020	0.233	0.073	0.051	0.206
14.60566	0.188	0.147	0.001	0.033	0.802	0.135	0.495	0.245	0.152	0.117	0.025	0.040	0.153	0.113	0.088	0.253
17.85136	0.392	0.153	0.021	0.050	0.831	0.140	0.082	0.259	0.139	0.137	0.045	0.052	0.127	0.134	0.113	0.266
17.85136	0.392	0.153	0.021	0.050	0.831	0.140	0.082	0.259	0.139	0.137	0.045	0.052	0.127	0.134	0.113	0.266
21.09706	0.412	0.136	0.160	0.070	0.806	0.125	0.192	0.204	0.126	0.119	0.073	0.066	0.113	0.120	0.129	0.211
24.34277	0.585	0.196	0.534	0.086	0.846	0.185	0.269	0.304	0.123	0.212	0.093	0.079	0.109	0.208	0.142	0.309
25.96562	0.564	0.163	0.704	0.090	0.819	0.152	0.261	0.229	0.127	0.172	0.101	0.081	0.112	0.171	0.144	0.234
27.58847	0.511	0.172	0.778	0.092	0.727	0.162	0.262	0.220	0.127	0.187	0.106	0.080	0.113	0.184	0.147	0.224
30.83417	0.421	0.160	0.888	0.091	0.545	0.150	0.215	0.187	0.114	0.174	0.118	0.073	0.099	0.174	0.146	0.190
34.07987	0.289	0.150	0.715	0.088	0.381	0.139	0.119	0.218	0.091	0.166	0.131	0.062	0.076	0.165	0.138	0.220
42.19413	0.248	0.025	0.270	0.067	0.794	0.025	0.076	0.160	0.043	0.028	0.107	0.049	0.032	0.035	0.096	0.165
50.30838	0.473	0.025	0.345	0.119	0.967	0.024	0.050	0.236	0.065	0.049	0.178	0.084	0.046	0.048	0.100	0.251
58.42264	0.626	0.033	0.873	0.165	0.779	0.031	0.067	0.064	0.058	0.011	0.222	0.130	0.037	0.033	0.121	0.066
66.53689	0.357	0.027	0.808	0.182	0.543	0.025	0.906	0.078	0.044	0.010	0.234	0.147	0.024	0.033	0.126	0.074
74.65115	0.144	0.024	0.772	0.177	0.257	0.024	0.856	0.092	0.038	0.009	0.218	0.147	0.021	0.032	0.127	0.087
82.7654	0.043	0.025	0.568	0.140	0.099	0.026	0.552	0.102	0.035	0.012	0.164	0.123	0.023	0.032	0.120	0.099
90.87966	0.025	0.034	0.316	0.123	0.065	0.034	0.407	0.107	0.047	0.017	0.136	0.117	0.035	0.034	0.107	0.108
98.99391	0.023	0.042	0.163	0.097	0.093	0.041	0.394	0.104	0.055	0.023	0.101	0.098	0.045	0.035	0.094	0.106
107.1082	0.060	0.038	0.189	0.090	0.175	0.038	0.381	0.091	0.050	0.020	0.098	0.089	0.042	0.031	0.080	0.095

Figure S28. ANOVA—Curvature parameters—both factors together.

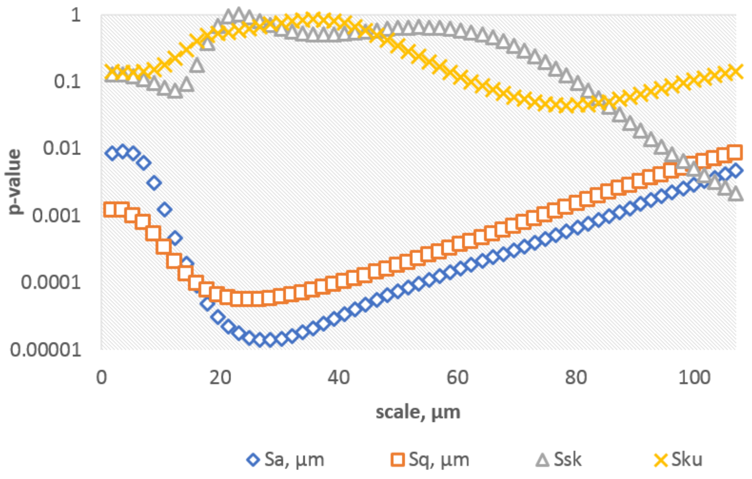

Figure S29. ANOVA—wavelet transformation- factor 1 - build direct.

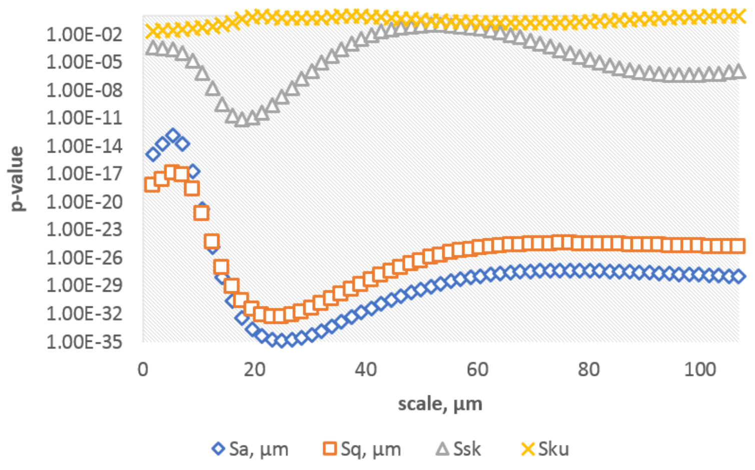

Figure S30. ANOVA—wavelet transformation - factor 2 - machining.

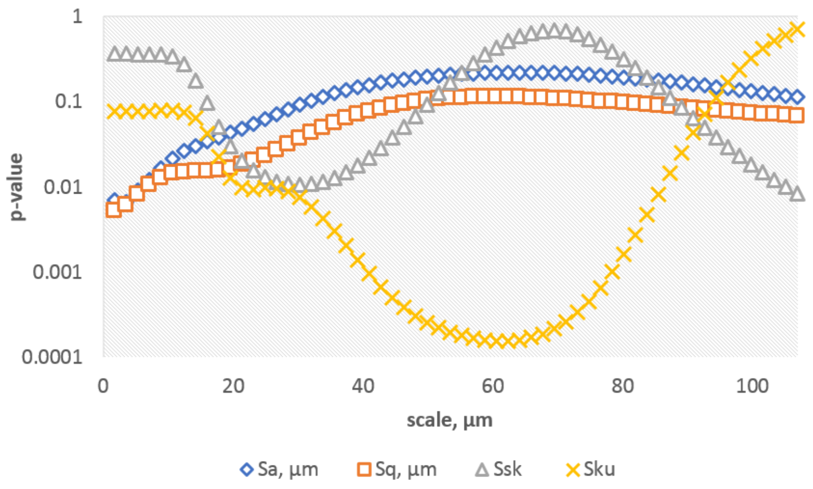

Figure S31. ANOVA—wavelet transformation - both factors together.
